# Genetic variation in an ephemeral mudflat species: The role of the soil seed bank and dispersal in river and secondary anthropogenic habitats

**DOI:** 10.1002/ece3.6109

**Published:** 2020-03-17

**Authors:** Jörg Böckelmann, Karin Tremetsberger, Kateřina Šumberová, Gudrun Kohl, Heinrich Grausgruber, Karl‐Georg Bernhardt

**Affiliations:** ^1^ Department of Integrative Biology and Biodiversity Research Institute of Botany University of Natural Resources and Life Sciences Vienna Austria; ^2^ Department of Vegetation Ecology Institute of Botany The Czech Academy of Sciences Brno Czech Republic; ^3^ Division of Plant Breeding Department of Crop Sciences University of Natural Resources and Life Sciences Vienna Austria; ^4^ Present address: Division of Tropical Ecology and Animal Biodiversity Department of Botany and Biodiversity Research University of Vienna Vienna Austria; ^5^ Present address: Division of Microbial Ecology Department of Microbiology and Ecosystem Science University of Vienna Vienna Austria

**Keywords:** *Cyperus fuscus*, Isoëto‐Nanojuncetea, long‐distance dispersal, microsatellites, ornithochory, selfing

## Abstract

Many ephemeral mudflat species, which rely on a soil seed bank to build up the next generation, are endangered in their natural habitat due to the widespread regulation of rivers. The aim of the present study was to elucidate the role of the soil seed bank and dispersal for the maintenance of genetic diversity in populations of near‐natural river habitats and anthropogenic habitats created by traditional fish farming practices using *Cyperus fuscus* as a model. Using microsatellite markers, we found no difference in genetic diversity levels between soil seed bank and above‐ground population and only moderate differentiation between the two fractions. One possible interpretation is the difference in short‐term selection during germination under specific conditions (glasshouse versus field) resulting in an ecological filtering of genotypes out of the reservoir in the soil. River populations harbored significantly more genetic diversity than populations from the anthropogenic pond types. We suggest that altered levels and patterns of dispersal together with stronger selection pressures and historical bottlenecks in anthropogenic habitats are responsible for the observed reduction in genetic diversity. Dispersal is also supposed to largely prohibit genetic structure across Europe, although there is a gradient in private allelic richness from southern Europe (high values) to northern, especially north‐western, Europe (low values), which probably relates to postglacial expansion out of southern and/or eastern refugia.

## Graphical abstract

Using microsatellite markers, we found no difference in genetic diversity levels between soil seed bank and above‐ground population and only moderate differentiation between the two fractions in the mudflat species *Cyperus fuscus*, possibly as a result of differences in short‐term selection during germination under specific conditions (glasshouse versus field) resulting in an ecological filtering of genotypes out of the reservoir in the soil. River populations, however, harboured significantly more genetic diversity than populations from fishponds and fish storage ponds, supposedly because of differences in the strength of selection pressures, historical bottlenecks, and altered levels and patterns of dispersal.

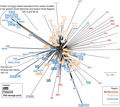

## 1 INTRODUCTION

The spatial separation of populations influencing rates of gene flow among populations was already a key element in early concepts and models of population ecology and genetics (Wright, 1931, 1943). The metapopulation model, which has been successfully applied to many short‐lived mobile animals (mostly invertebrates), describes how discrete local populations that are present temporally in highly fragmented habitat patches are connected through migration. According to the metapopulation model, local extinctions and (re‐)colonizations are recurrent rather than unique events (Hanski & Gilpin, 1997), especially in habitats with high levels of physical disturbance. Migration in the form of seeds dispersing to suitable habitat patches is a fundamental component of plant metapopulations. A prominent example of a plant metapopulation is provided by the annual emergent aquatic plant *Eichhornia paniculata*, which occurs in transient pools in north‐eastern Brazil (Barrett & Husband, 1997; Husband & Barrett, 1998). The species does not maintain a permanent soil seed bank. It is thought that seeds are dispersed by birds and cattle as well as through flash floods in the rainy season. So far, however, accounts of plant metapopulations remain scarce (e.g., Freckleton & Watkinson, 2002; Honnay, Jacquemyn, Looy, Vandepitte, & Breyne, 2009).

The presence of a soil seed bank (buried viable seeds) complicates the metapopulation model in plants, since recruitment from the soil seed bank can be thought of as dispersal through time (Freckleton & Watkinson, 2002; McCauley, 2014). The evolutionary consequences of the soil seed bank are manifold. It may function as a “genetic memory” by storing genetic variability and hence local adaptation to habitat in viable seeds (Honnay, Bossuyt, Jacquemyn, Shimono, & Uchiyama, 2008; Mandák, Zákravský, Mahelka, & Plačková, 2012). The soil seed bank of annual plants may eliminate the selective impact of environmental conditions that fluctuate randomly from year to year and may retard the response to constant selection (Levin, 1990; Templeton & Levin, 1979). By increasing the effective population size, the soil seed bank may also protect populations from genetic drift (Lundemo, Falahati‐Anbaran, & Stenøien, 2009; Nunney, 2002). Moreover, a high dispersal rate in time may partly counteract the homogenizing effect of spatial dispersal, as it has been shown in the annual ruderal plant *Arabidopsis thaliana* (Falahati‐Anbaran, Lundemo, & Stenøien, 2014).

Persistent soil seed banks with seeds as long‐lived as 50‒100 years resulting in a complex age structure of the soil seed bank are characteristic of ephemeral (annual), fugitive species (Leck, 1989; Levin, 1990). Muddy shorelines of lakes and rivers (mudflats), which are characterized by an intensive disturbance regime of changing water levels usually providing exposed ground only in late summer, shelter a highly specialized vegetation of wetland annuals with very short development cycles from germination to reproduction, high seed production, and rapidly germinating seeds lacking inborn dormancy (communities of dwarf rushes of the class Isoëto‐Nanojuncetea). A key life history trait of mudflat species is the maintenance of a persistent soil seed bank, with which the species survive flooded, and so unsuitable, periods (Baskin & Baskin, 2014; von Lampe, 1996). Dormancy is forced by external factors such as darkness, lack of oxygen, and lack of temperature fluctuations during flooding (Leck, 1989). Hydrochory, ornithochory, and ichthyochory are likely dispersal strategies of these wetland plants (Figuerola & Green, 2002; Soons, Vlugt, Lith, Heil, & Klaassen, 2008; VonBank, DeBoer, Casper, & Hagy, 2018).

Regulation of the majority of the world's rivers has led to a dramatic loss of floodplain habitats (Grass, Tremetsberger, Hössinger, & Bernhardt, 2014; Hein et al., 2016). Natural habitats in lakes suffer from eutrophication as well as hydrodynamic and management changes (Mørk, Kragh, Kristensen, & Sand‐Jensen, 2018; Wantzen et al., 2008). Today, possible secondary anthropogenic habitats of threatened wetland species are therefore crucial for the conservation of biodiversity. Fishpond and fish storage pond systems with a historical Central European distribution hotspot in the Czech Republic are maintained by fish farms. They provide a rich mosaic of different wetland habitats with relatively natural features suitable as substitute habitats for threatened mudflat species (Francová, Šumberová, Janauer, & Adámek, 2019; Květ, Jeník, & Soukupová, 2002; Richert et al., 2016; Šumberová, Ducháček, & Lososová, 2012; Wezel et al., 2014). Nevertheless, the evolutionary forces that shape the genetic structure of species may be altered in anthropogenic versus natural habitats, which may provide opportunities for adaptive niche shifts (Kamdem et al., 2012). Divergent environmental conditions such as the frequency and intensity of flooding during plant growth are expected to exert divergent selection pressures in river and anthropogenic wetland habitats, which may lead to phenotypic differentiation (Böckelmann, Tremetsberger, Šumberová, Grausgruber, & Bernhardt, 2017). Genetic and/or epigenetic differentiation could underlie such differential adaptation (Bossdorf, Richards, & Pigliucci, 2008). Furthermore, the level of gene flow among sites by hydrochorous, ornithochorous, or ichthyochorous seed dispersal may be markedly different in the anthropogenic habitats.

Little is known about the genetic structure of mudflat species. We studied neutral genetic variation in *Cyperus fuscus* L., which is a common representative of mudflat habitats, using microsatellite markers. The following questions were in the focus of our study: (a) Does the persistent soil seed bank function as a “genetic memory”? If the seed bank really worked as a genetic memory, we would expect higher genetic diversity in the soil. Does the genetic composition of the above‐ground population differ from that of the soil seed bank? If selection acted as a filter on the alleles present in the soil during germination and growth of the above‐ground individuals, we would expect genetic differentiation between the seed bank and the above‐ground population. (b) Do populations from river and anthropogenic habitats differ in their levels of within‐populational variation? We would expect river and anthropogenic populations to differ, because they are exposed to different habitat conditions, which in turn influence determining factors of within‐populational variation such as dispersal, drift, and selection. Are the populations from river and anthropogenic habitats genetically differentiated? If they were exposed to divergent selection pressures, we would expect them to be genetically differentiated. (c) The first two questions were studied in Central Europe, but *Cyperus fuscus*, as many other mudflat species, occurs sporadically all over Europe—for short periods of time and at different points in time—meaning that populations are isolated spatially and temporally from each other. Our third question therefore was whether populations across a larger geographic area are genetically differentiated. If the action of drift and/or selection was stronger than gene flow, we would expect genetic differentiation. Moreover, we wanted to know whether European populations differ in levels of within‐populational variation. If current and/or previous environmental conditions better supported the survival of *Cyperus fuscus* in some regions, we would expect higher levels of variation in these regions.

## 2 MATERIALS AND METHODS

### 2.1 Study species and habitats

*Cyperus fuscus* is an annual, self‐compatible, and highly plastic graminoid native to the Mediterranean and temperate Eurasia (Böckelmann et al., 2017; East, 1940). It is a typical mudflat species growing on muddy, sandy, or gravelly substrata on mudbanks of rivers, lakes, pools, and ponds, which are usually exposed in later summer (class Isoëto‐Nanojuncetea; Hejný, 1960). It also occurs in historical and extensively or semi‐intensively used fishponds in Bohemia and other regions of Europe, which provide secondary habitats for many mudflat species (Květ et al., 2002; Richert et al., 2016). Fish storage ponds are small basins with a sandy, stony, or clayey ground in which fish is stored alive after harvest. Most of the year, they are not filled with water, but some mudflat species such as *Cyperus fuscus* can tolerate these rather unnatural conditions (Šumberová, Ducháček, et al., 2012; Šumberová, Lososová, Fabšičová, & Horáková, 2006). As in other mudflat species, the yearly above‐ground populations are derived from a persistent soil seed bank (Baskin & Baskin, 2014; Thompson, Bakker, & Bekker, 1997). The small achenes have no particular dispersal features and are supposedly dispersed by gusts of wind (boleochory), running water (hydrochory), as well as animals and humans, for example, in mud adhering to waterfowl or rubber boots of fish farmers (ornithochory and anthropochory; Müller‐Schneider, 1986; Šumberová & Ducháček, 2017; Šumberová, Ducháček, et al., 2012; Šumberová, Lososová, Ducháček, Horáková, & Fabšičová, 2012; von Lampe, 1996). Dispersal by fish (ichthyochory) may be another dispersal mode (K. Šumberová, unpubl. data).

### 2.2 Sampling

**Figure 1:**
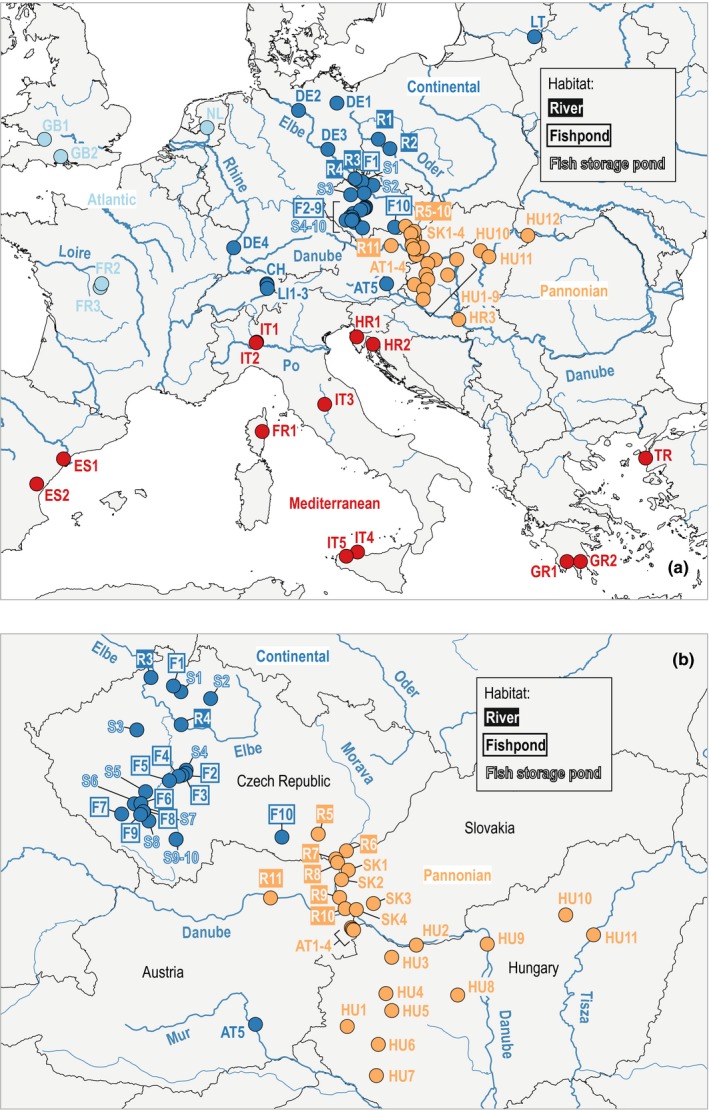
(a) Map of sampled populations across Europe: Red, Mediterranean region; orange, Pannonian region; dark blue, Continental region; light blue; Atlantic region. The three habitat types studied in Central Europe [rivers (R), fishponds (F), fish storage ponds (S)] are indicated by different text style. (b) Detailed map of populations sampled in Austria, the Czech Republic, Hungary, and Slovakia. Maps prepared in QGIS version 2.14 “Essen” (https://qgis.org/en/site/). See Appendix S1 for a list of population codes with collection information

We studied 31 populations belonging to one of the three habitat types (rivers, fishponds, and fish storage ponds) in Central Europe and 49 additional populations across Europe. The localities were roughly assigned to one of four coarse biogeographical regions (Mediterranean, Pannonian, Continental, and Atlantic regions; European Environment Agency, 2017; Figure 1; Appendix S1). The delimitation of the Pannonian region in Austria followed Niklfeld (1964). Vouchers of most populations were deposited in the herbarium of the University of Natural Resources and Life Sciences, Vienna (Index Herbariorum code: WHB; http://sweetgum.nybg.org/science/ih/).

The populations of the three habitat types are the same as those used for the environmental manipulation experiment of Böckelmann et al. (2017). For each of these populations, soil samples from two depths [0–5 cm deep soil (shallow seed bank) and 5–15 cm deep soil (deep seed bank)] and ripe seeds were collected from three plots per locality in summer 2012 and stored in the dark at 6°C for approximately six months. Exceptionally, seeds of above‐ground individuals were collected from throughout the population, without consideration of the plots, in the localities R11 and S3. In April 2013, the soil samples of the same soil fraction and plot within a locality were pooled and carefully homogenized before they were spread on sterile sand. Emerging seedlings of *Cyperus fuscus* were pricked and individually grown in plastic pots. Later, leaf samples were taken and dried on silica gel. Similarly, the ripe seeds collected from above‐ground individuals in the field were seeded and leaf samples from the grown plants were put into silica gel. More details of the sampling procedure are given in Böckelmann et al. (2017). Leaf samples of the additional populations across Europe were taken in the field, where they were immediately put into bags with silica gel for drying.

### 2.3 Microsatellite genotyping

Genomic DNA of the silica gel‐dried leaf samples was extracted with the DNeasy Plant Mini Kit (QIAGEN, Hilden, Germany) following the manufacturer's instructions. We amplified 21 polymorphic microsatellite loci previously isolated from *Cyperus fuscus* following the method detailed in Böckelmann, Wieser, Tremetsberger, Šumberová, and Bernhardt (2015). In total, 1,444 individuals were analyzed on a 3,500 Genetic Analyzer (Applied Biosystems) and genotyped using GeneMarker version 2.4 (SoftGenetics). Allelic data were treated as standard genotypic data and coded by their length in base pairs.

### 2.4 Analyses of genetic variation within and among the above‐ground and soil fractions of populations from the three habitat types in Central Europe

#### 2.4.1 Genetic diversity

For the analysis of genetic diversity within the soil and above‐ground fractions of populations, only fractions of populations with at least five amplified individuals were considered. Because it was not possible to get five or more unambiguously genotyped individuals from both soil fractions from every site (Appendix S2), individuals that originated from the shallow and deep soil seed bank of the same population were pooled for the analysis of genetic diversity. Observed heterozygosity (*H*_I_), expected heterozygosity under the Hardy–Weinberg equilibrium (*H*_S_), and the inbreeding coefficient (*F*_IS_) were calculated using GenAlEx version 6.5 (Peakall & Smouse, 2012). Because of the different sample sizes, the rarefaction method implemented in the program HP‐Rare version 1.1 (Kalinowski, 2005) was used to estimate allelic richness (*A*_r_) and private allelic richness (*pA*_r_) based on 10 randomly sampled alleles. Because we wanted to put *pA*_r_ of the above‐ground fraction in relation to that of the soil fraction, we used only the above‐ground and the soil fractions of a specific population as input for the program, omitting all other populations. This was done separately for each population. Differences in genetic diversity estimates were evaluated by means of linear mixed models using the procedure MIXED of SAS version 9.4 (SAS Institute, Cary, North Carolina, USA). Fraction, habitat as well as their interaction were used as fixed factors. Differences between least square means were tested for significance by Tukey–Kramer post hoc tests.

#### 2.4.2 Analysis of recent migration

To detect first‐ and second‐generation migrants from the genotype data, we performed Bayesian inference of recent migration using BayesAss version 3.0.4 (Wilson & Rannala, 2003), whereby we coded all fractions and sampling plots within localities as the same source population. We also combined populations F6‒9 and S6‒8 as well as S9‒10 into only two discrete source populations as these are each geographically and genetically very close (see Figures 1, 4). PGDSpider version 2.1.1.5 (Lischer & Excoffier, 2012) was used to convert our dataset into the IMMANC format used by BayesAss. Ten independent runs with 10 million iterations (discarding the first 5 million iterations and sampling every thousandth iteration) and different seeds for the random number generator were carried out. The mixing parameter for allele frequencies was adjusted to 0.8 and that for inbreeding coefficients to 0.6 to achieve a proper mixing of the Markov chain Monte Carlo (MCMC) analysis. Convergence was checked in Tracer version 1.7.1 (Rambaut, Drummond, Xie, Baele, & Suchard, 2018). We then calculated the mean across the ten runs of the posterior probabilities of ancestry of the 985 individuals of the populations from the three habitat types in Central Europe. Posterior probabilities of ancestry of first‐ and second‐generation migrants from the same source population were summed for each individual. The expected share of residents and immigrants was calculated for populations or fractions within populations from the individual posterior probabilities of ancestry.

#### 2.4.3 Genetic differentiation and structure

To assess genetic differentiation among fractions within populations (above‐ground, shallow seed bank, and deep seed bank) as well as among the three sampling plots within populations, we conducted analyses of molecular variance (AMOVA) in Arlequin version 3.5 (Excoffier & Lischer, 2010). The calculations were performed locus by locus, and all individuals were included, independent of their level of missing data (allowed missing level per site = 1). The analysis was not carried out for populations F4, F7–F9, and S6–S8 with very low genetic diversity values, that is, a mean expected heterozygosity across all (soil seed bank and above‐ground) individuals of less than or equal to 0.05. Populations R11 and S3 had to be omitted from this analysis, because the above‐ground individuals were sampled outside of the plots, from which the soil samples had been taken. AMOVA were also carried out for all populations of the three habitat types in Central Europe to assess genetic differentiation among habitat types, biogeographical regions, and river systems, whereby all fractions and plots within localities were coded as the same population. The grouping of populations followed Appendix S2.

To display genetic relationships among all populations analyzed, a population pairwise *F*_ST_‐matrix (generated in Arlequin with allowed missing level per site = 1) was used to produce a Neighbor‐Net network with SplitsTree4 version 4.14.5 (Huson & Bryant, 2006). To this end, populations of the three habitat types in Central Europe were divided into two fractions, soil seed bank (shallow and deep soil fractions taken together) and above‐ground population.

### 2.5 Analyses of genetic variation within and among populations across Europe

#### 2.5.1 Genetic differentiation

AMOVA were carried out for populations across biogeographical regions in Europe to assess genetic differentiation among biogeographical regions and river systems. Only individuals of the above‐ground fractions of the populations from the three habitat types in Central Europe entered in these analyses and—throughout the European scale—only river systems with at least two sampled populations were considered. First, all populations that could be unambiguously assigned to one of six large river systems (Danube, Elbe, Oder, Rhine, Loire, Po) were included (regardless of their level of human impact and proximity to running water). The populations were grouped according to Appendices S2 and S3 [population IT2 and populations HU6 and HU9 (not in Appendix S3) were assigned to the river systems Po and Danube, respectively]. Second, all populations analyzed were included to assess differentiation among biogeographical regions. The analyses were carried out with the same settings as before.

#### 2.5.2 Genetic isolation by distance

We analyzed the relationship between population pairwise genetic distance (*F*_ST_; as above) and geographic (linear) distance (generated from the population coordinates in GenAlEx) between all populations analyzed (using only individuals of the above‐ground fraction of the populations from the three habitat types in Central Europe) in R version 3.5.1 (R Core Team, 2018; function cor.test). A linear model was fitted to obtain the regression coefficient (functions lm and predict). A Mantel correlogram was used to assess the extent of spatial autocorrelation across Europe using the same matrices as input (function mantel.correlog of the R‐package vegan version 2.5–2; Oksanen et al., 2018). 9,999 permutations were requested for significance testing, and the Holm progressive correction for multiple testing was applied.

#### 2.5.3 Genetic diversity

*H*_I_, *H*_S_, *F*_IS_, *A*_r_, and *pA*_r_ were estimated for all populations with at least five amplified individuals (using only the above‐ground fractions of all populations) with the same settings as before, but *pA*_r_ was estimated for all populations across Europe in a single step. Because *pA*_r_ is dependent on the density of the sampled populations, which differs considerably between biogeographical regions, we also estimated it after arbitrarily thinning out populations (two arbitrary selections of populations), so that only four populations remained in each region. Differences in genetic diversity estimates were evaluated by means of linear mixed models with the same settings as before using biogeographical region as fixed factor. To better understand genetic diversity in the biogeographical regions, *A*_r_ and *pA*_r_ were also estimated for the regions as a whole (i.e., treating each region as if it was a population) using rarefaction analysis with 22 randomly sampled alleles per region. In order to balance the geographic area occupied by populations in a biogeographical region between the regions, *A*_r_ and *pA*_r_ were also evaluated by rarefaction analysis for just two regions as a whole (the Mediterranean region on the one hand and the Pannonian, Continental, and Atlantic regions lumped into a single area on the other hand; 210 randomly sampled alleles per region).

## 3 RESULTS

**Figure 2:**
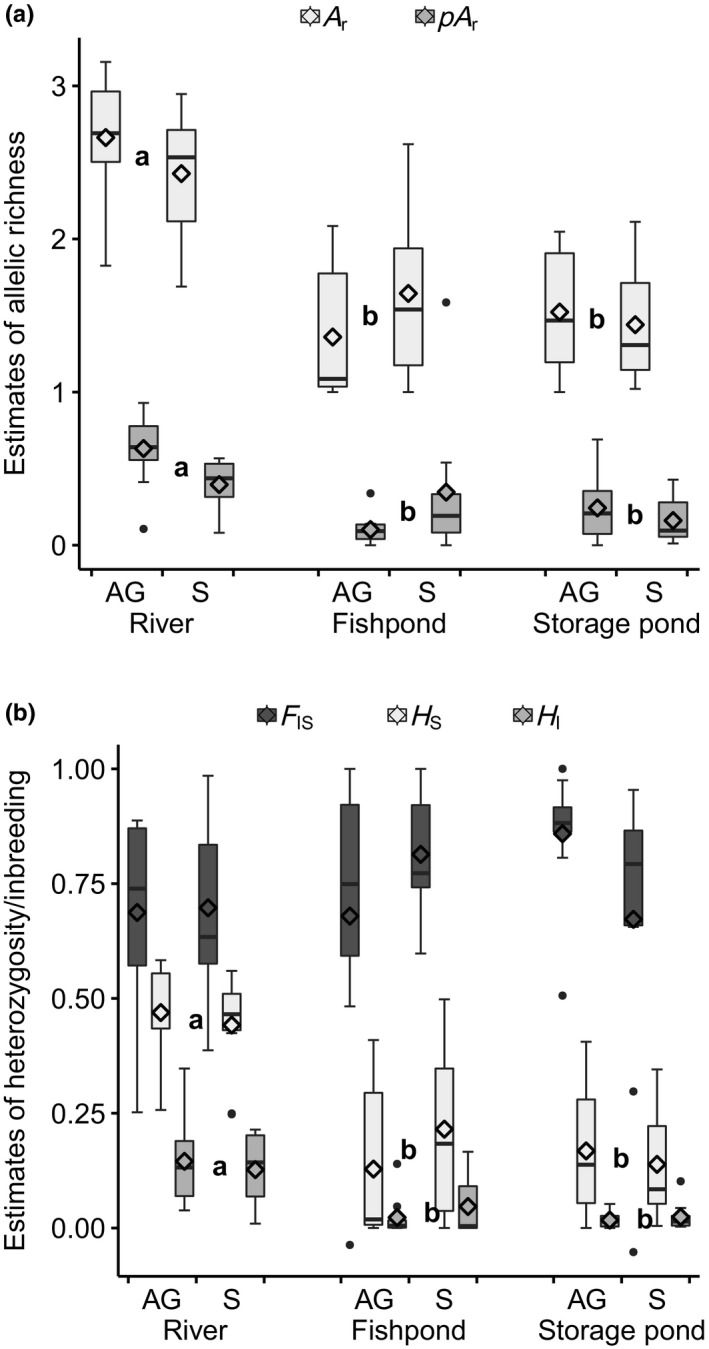
Distribution of genetic diversity estimates of populations from rivers, fishponds, and fish storage ponds (Appendices S2). Boxplots with mean points of the above‐ground (AG) and soil (S) fractions are shown for each habitat type: (a) allelic richness (*A*_r_) and private allelic richness (*pA*_r_); (b) observed and expected heterozygosity (*H*_I_, *H*_S_) and the inbreeding coefficient (*F*_IS_). Letters denote belonging of habitat types to significantly different groups according to linear mixed modeling (Tukey–Kramer adjustment for multiple comparisons: *p* < .05; Appendix S4)

**Figure 3:**
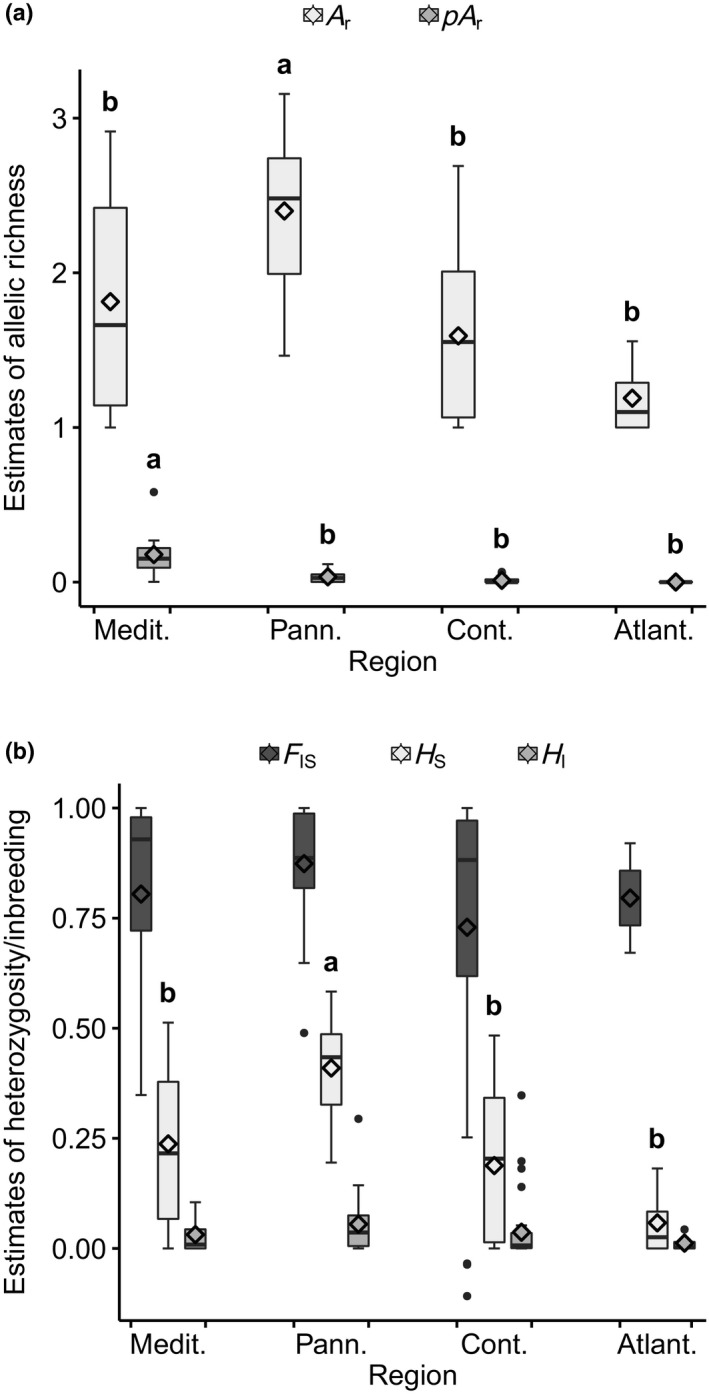
Distribution of genetic diversity estimates (boxplots with mean points) of populations from different biogeographical regions in Europe (Appendix S3): (a) allelic richness (*A*_r_) and private allelic richness (*pA*_r_); (b) observed and expected heterozygosity (*H*_I_, *H*_S_) and the inbreeding coefficient (*F*_IS_). Only the above‐ground fractions of populations, for which the soil seed bank had also been sampled, entered in this graph. Letters denote significant differences in least square means according to linear mixed modeling (Tukey–Kramer adjustment for multiple comparisons: *p* < .05; Appendix S4)

All 21 microsatellite markers were polymorphic in our sample. None of the scored plants had more than two distinct alleles per marker, so that all plants were interpreted to be diploid. The number of alleles per marker in the entire sample ranged from 5 to 40 with a mean of 12.4. There were 260 different alleles altogether. Observed heterozygosity was usually considerably lower than expected heterozygosity. This explains the high inbreeding coefficients (Figures 2 and 3; Appendices S2 and S3).

### 3.1 Genetic diversity and differentiation of the above‐ground and soil fractions

No significant differences were detected in genetic diversity estimates between the above‐ground and the soil fraction in all three habitat types (Figure 2; Appendix S4).

Inspection of the trace of the log likelihood of the analysis of recent migration showed that it somehow stabilized after ~ 4‒5 million iterations. Of course, one must keep in mind that only a small fraction of potential source populations, namely the localities analyzed here, have been sampled. For this reason, one cannot interpret the results as actual dispersal events from a particular source population, but rather as indications of the general region of origin of immigrants. The proportion of immigrants into the above‐ground and soil fractions of populations did not differ significantly in all three habitat types (Table1).

**Table 1:** Mean and standard deviation of the proportion of first‐ and second‐generation immigrants into the above‐ground and soil fractions of populations in various habitat types calculated by BayesAss

Habitat/fraction	*N*_p_	Mean ± *SD* of the proportion of immigrants into…
River populations		
… the above‐ground population	11	37.4 ± 25.8
… the shallow and deep soil seed bank	11	40.3 ± 28.0
Probability of *t* test		0.613
Fishpond populations		
… the above‐ground population	10	10.4 ± 19.6
… the shallow and deep soil seed bank	10	29.2 ± 37.5
Probability of *t* test		0.176
Fish storage pond populations		
… the above‐ground population	10	18.4 ± 30.2
… the shallow and deep soil seed bank	10	20.7 ± 32.6
Probability of *t* test		0.705

Note

The probabilities of paired *t* tests with two‐sided distributions (testing whether there is a difference in the proportion of immigrants into the above‐ground population or the soil seed bank, respectively) are shown for each habitat type. *N*_p_, number of populations for calculation of the mean and standard deviation.

The values of differentiation among fractions within populations were in a similar range as among sampling plots within populations (Table2). The high value of the 75% quartile of the value of differentiation among fractions within fishpond populations goes back to populations F6 and F10, with values of differentiation of 63.3% and 73.4%, respectively.

**Table 2:** Analyses of Molecular Variance (AMOVA) within populations of *Cyperus fuscus*

Grouping	*N*_p_	Median and interquartile range of the mean percentage of variation…
… among fractions within populations		
River populations	10	7.6 (4.5–10.9)
Fishpond populations	6	6.8 (3.0–65.8)
Fish storage pond populations	6	7.7 (4.1–15.2)
… among sampling plots within populations		
River populations	10	6.8 (4.5–12.5)
Fishpond populations	6	11.4 (3.6–18.3)
Fish storage pond populations	6	9.2 (6.1–13.1)

Note

The median and interquartile range across populations of the mean percentage of variation among fractions (above‐ground, shallow soil seed bank, deep soil seed bank) and among sampling plots is shown. *N*_p_, number of populations for calculation of the median and interquartile range.

**Figure 4:**
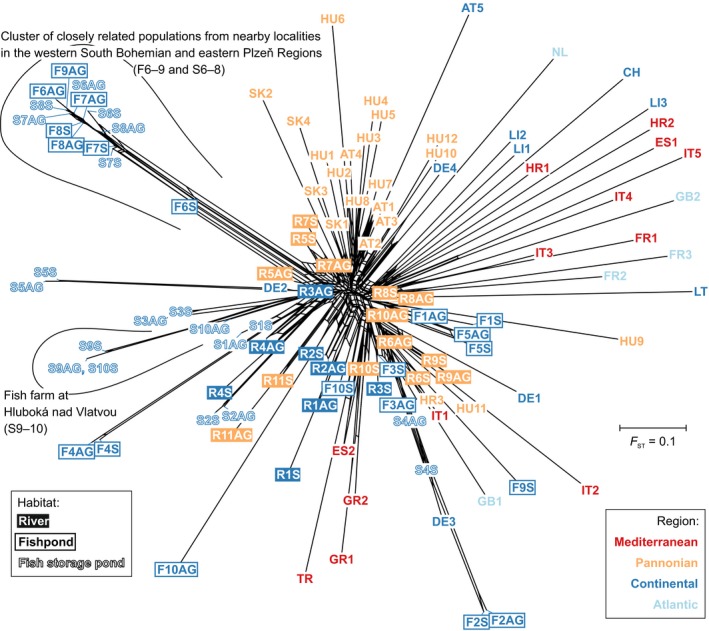
Neighbor‐Net network based on genetic distances (*F*_ST_) showing relationships among populations of the three habitat types in Central Europe and across biogeographical regions in Europe. AG, above‐ground population; S, soil seed bank. See Figure 1 for a map of populations and Appendix S1 for a list of population codes with collection information

The Neighbor‐Net network shows the differentiation of populations and fractions within populations based on the *F*_ST_ distance matrix (Figure4). In most cases, the above‐ground population and the soil seed bank from the same locality are situated in proximity on the network.

### 3.2 Genetic diversity and differentiation of populations in river and anthropogenic habitats

River populations had significantly higher values in genetic diversity estimates (*A*_r_, *pA*_r_, *H*_I_, and *H*_S_) than populations from the two anthropogenic habitats, and the latter did not differ significantly (Figure 2; Appendix S4).

**Figure 5:**
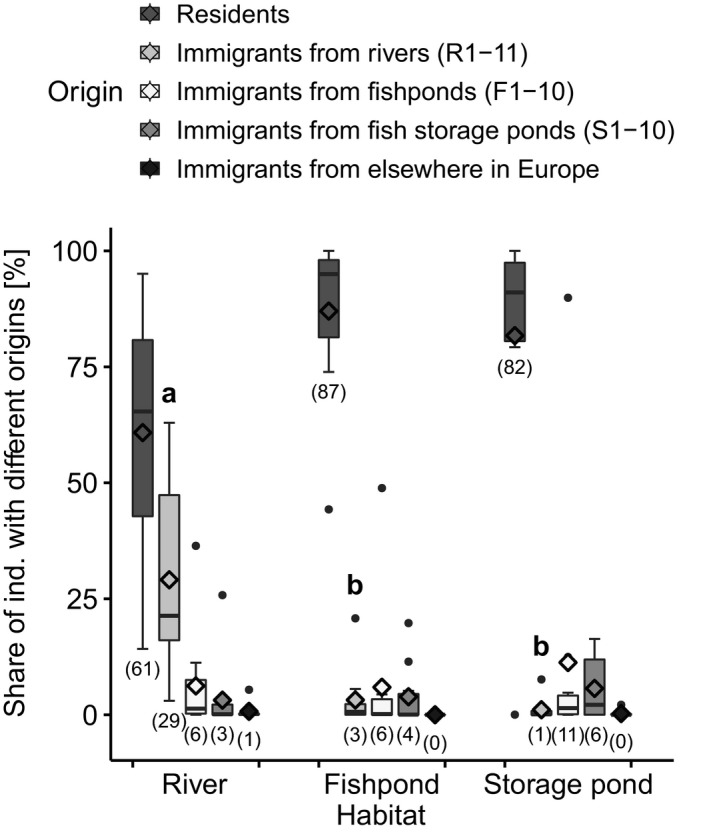
Summary results of the analysis of recent migration with BayesAss. Boxplots with mean points of the expected share of residents as well as of first‐ and second‐generation immigrants into populations are shown (mean values in parenthesis). Immigrants were classified according to their origin: from river, fishpond, or fish storage pond habitats in Central Europe or from elsewhere in Europe. Letters denote significant differences in least square means according to linear mixed modeling (Tukey–Kramer adjustment for multiple comparisons: *p* < .001)

The summary results of the analysis of recent migration (Figure5) showed that river populations received significantly more immigrants from other river localities (29%) than fishpond and fish storage pond populations, which received just 3% and 1% immigrants from river localities, respectively. The mean posterior probability of an individual of being an immigrant (corresponding to the expected share of immigrants in the population) multiplied by linear dispersal distance was also significantly larger for river populations (3,898; *SD* = 3,128) than for fishpond populations (1569; *SD* = 1,261) or fish storage pond populations (1,432; *SD* = 830; *p* < .05). The mean expected percentage share of immigrants from up‐ or downstream with respect to total migration along the river system [considering the rivers Oder (R1–2), Elbe (R3–4) and Danube with its tributaries (R5–11)] was 51.3 (*SD* = 8.4) for immigrants from upstream and 48.7 (*SD* = 8.4) for immigrants from downstream.

AMOVA revealed a moderate variation of 8.1% among the three habitat types (Table3). Most variation was detectable within populations (49.4%) and among populations within the same habitat type (42.5%). Differentiation among biogeographical regions (7.6%) and river systems (9.8%) was in a similar range as among habitat types.

**Table 3:** Summary results of analyses of molecular variance (AMOVA) of *Cyperus fuscus*

Grouping	*N*_p_	*N*_i_	*df*	Mean percentage of variation among groups
Populations of the three habitat types in Central Europe				
Habitat: rivers, fishponds, fish storage ponds	31	985	2	8.1 (5.7–10.6)
Biogeographical region: Pannonian, Continental	31	985	1	7.6 (4.5–11.2)
River system: Danube, Elbe, Oder	31	985	2	9.8 (6.4–13.4)
Populations of six large river systems				
Biogeographical region: Mediterranean, Pannonian, Continental, Atlantic	65	881	3	9.1 (6.4–11.9)
River system: Donau, Elbe, Oder, Rhine, Loire, Po	65	881	5	14.7 (11.7–18.3)
All populations across biogeographical regions in Europe				
Biogeographical region: Mediterranean, Pannonian, Continental, Atlantic	80	1,019	3	11.9 (8.9–15.4)

Note

The mean percentage of variation explained by various groupings is shown along with the 95% bootstrap percentile values. *N*_p_, number of populations; *N*_i_, number of individuals; *df*, degrees of freedom.

In the Neighbor‐Net network, populations from river and anthropogenic habitats are not substantially separated (Figure4). Most river populations are close to the center of the network, whereas most fishpond and fish storage pond populations are located on longer branches. One cluster of very closely related populations (F6–9 and S6–8) appears on a single, well‐defined branch (except for the sole individual sampled from the soil fraction of population F9, which was classified as a recent immigrant). The populations from two fish storage ponds within the same fish storage pond system are also on their own branch (S9–10).

### 3.3 Distribution of genetic diversity across Europe

AMOVA revealed that in the case of populations of the six large river systems (Danube, Elbe, Oder, Rhine, Loire, and Po, regardless of their level of human impact and proximity to running water), remarkable 14.7% variation was found among river systems compared with 9.1% variation among biogeographical regions (Table3). On the larger geographic scale (considering all populations across Europe), the differentiation among biogeographical regions was 11.9%.

The Neighbor‐Net network (Figure4) is “bush‐like” with usually long branches leading to single populations. Only a very coarse geographic pattern is visible.

**Figure 6:**
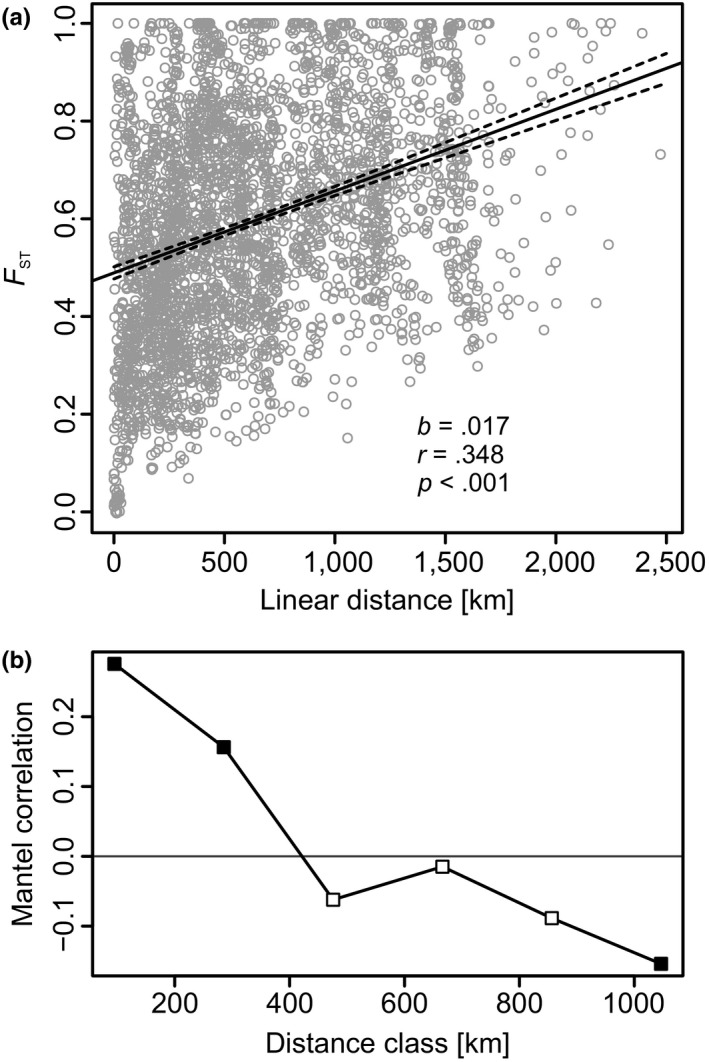
(a) Linear model with the 95% confidence interval on the fitted values of population pairwise genetic distance (*F*_ST_) in dependence of the linear distance of all populations analyzed. *b*, regression coefficient [*F*_ST_/100 km]; *r*, Pearson correlation coefficient; *p*, *p*‐value. (b) Mantel correlogram limited to the first six distance classes of populations of *Cyperus fuscus* across biogeographical regions in Europe showing significant spatial correlation after Holm correction for multiple testing of populations up to ~ 285 km distant (black squares: *p* < .05)

There is a significant pattern of isolation by distance across all populations analyzed in Europe (Figure6a). The Mantel correlogram (Figure6b) shows a significant positive spatial correlation of populations up to ~285 km distant (probability corrected for multiple testing = 0.0004), whereas populations in higher distance classes (~475 km or more) show a negative correlation.

**Figure 7:**
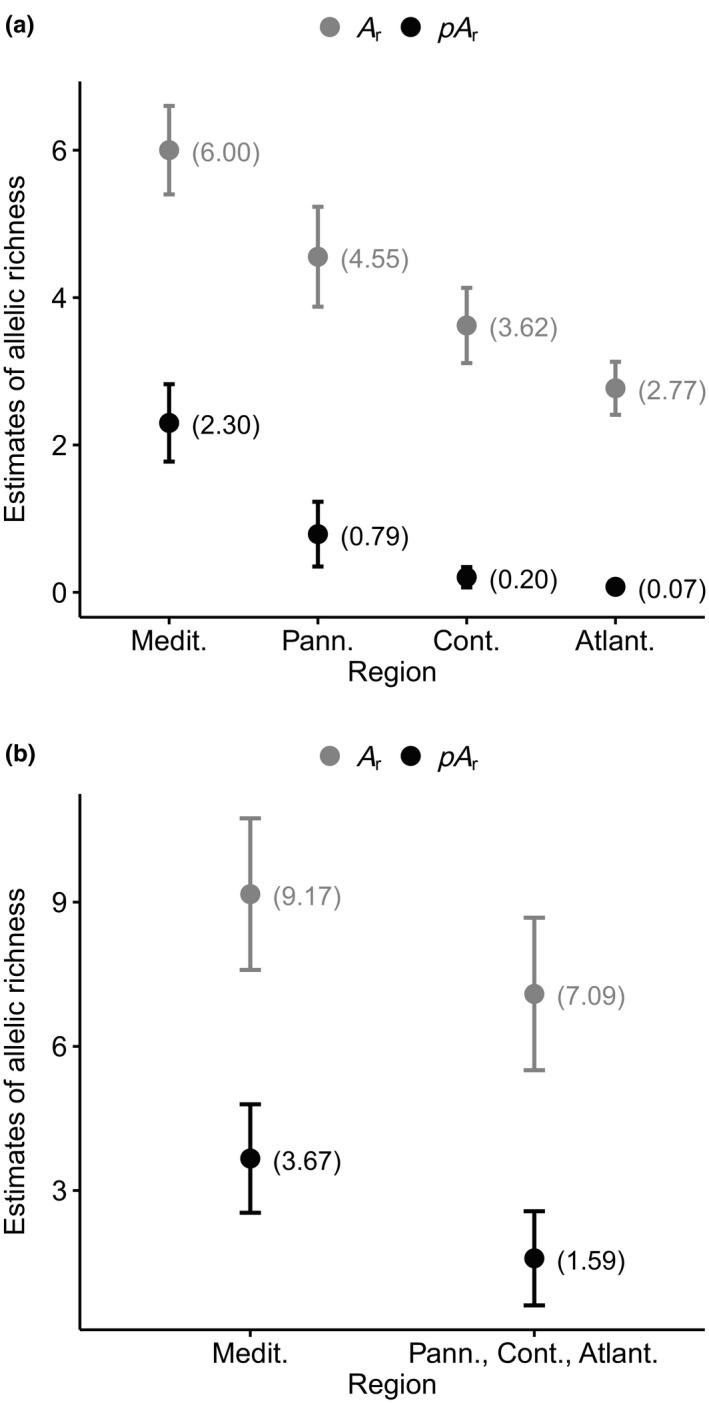
Mean (values in parenthesis) and 95% confidence interval over loci of allelic richness (*A*_r_) and private allelic richness (*pA*_r_) in biogeographical regions in Europe based on rarefaction analysis. In (a), 22 alleles were randomly sampled per region. In (b), the Pannonian, Continental, and Atlantic regions were lumped into a single area (210 randomly sampled alleles per region)

The Pannonian populations had significantly higher values in two genetic diversity estimates (*A*_r_ = 2.30, *H*_S_ = 0.41) than populations from the other biogeographical regions in Europe, whereas private allelic richness was highest in the Mediterranean populations (0.18; Figure 3; Appendix S4). The same order of decreasing private allelic richness in populations of the Mediterranean, Pannonian, Continental, and Atlantic regions is seen after thinning out populations to an equal number in each region (Appendix S5). The rarefaction analysis of *A*_r_ and *pA*_r_ based on 22 randomly sampled alleles per region showed even clearer results. Allelic richness and private allelic richness were highest in the Mediterranean region, second highest in the Pannonian region, and third highest in the Continental region. The Atlantic region had the lowest values (Figure7a). The alternative grouping with two regions confirmed this pattern. The Mediterranean region had higher allelic richness and private allelic richness than the Pannonian, Continental, and Atlantic regions taken together (Figure7b).

## 4 DISCUSSION

To the best of our knowledge, this is the first study of genetic diversity of a typical mudflat species. We found comparable values of genetic diversity within populations of *Cyperus fuscus* [mean expected heterozygosity in above‐ground populations across Europe = 0.27 (range = 0.00–0.58)] as reported for *Medicago lupulina*, another annual (to short‐lived perennial), self‐compatible plant with a soil seed bank [mean expected heterozygosity = 0.25 (range = 0.06–0.40); Yan, Chu, Wang, Li, & Sang, 2009; also based on microsatellite markers]. The inbreeding coefficient of *Cyperus fuscus* [mean = 0.80 (range = −0.11 to 1.00)] is slightly lower than the one reported for *Medicago lupulina* [mean = 0.92 (range = 0.74 to 1.00)], but—given the rather large population sizes (Appendix S1)—still suggests a high selfing rate.

### 4.1 The role of the soil seed bank for the genetic diversity of the above‐ground population

It has been hypothesized that seeds of many generations stored in the soil increase diversity and effective population size (Templeton & Levin, 1979; Zaghloul, Reisch, & Poschlod, 2013). For annual wetland plants, a much larger proportion of seeds normally remains in the soil than germinates (Bernhardt, Koch, Kropf, Ulbel, & Webhofer, 2008; Deil, 2005; Leck & Brock, 2000). However, we could not find any evidence for an accumulation of genotypes in the soil (Figure2). This supports previous findings of Honnay et al. (2008) and Mandák et al. (2012) that the soil seed bank does not harbor higher genetic diversity. One explanation in *Cyperus fuscus* might be high levels of immigration into both the soil seed bank and the above‐ground population (Table1). However, choice of technique is important for the analysis of the soil seed bank of ephemeral wetlands (Bernhardt et al., 2008; Price, Wright, Gross, & Whalley, 2010). In our experiment, just those individuals have been genotyped that germinated from the soil under glasshouse conditions, so the full amount of viable seeds in the soil stayed unclear. Some other genotypes may have remained stored in the soil, because the seeds did not germinate in cultivations. The filtering given by the cultivation conditions could explain why we did not find higher values for the genetic diversity estimates in the individuals derived from the soil.

Except for populations F6 and F10, the differentiation among fractions within populations was in a similar range as among the three sampling plots (Table2). We think that self‐thinning and short‐term selection acting as a filter on the alleles present in the soil during germination and growth of the above‐ground individuals could be responsible for the observed differentiation among fractions, as also suggested by Mandák, Bímová, and Plačková (2006) and Honnay et al. (2008). We interpret the differentiation among the sampling plots as small‐scale spatial heterogeneity within populations, which results from the predominantly selfing mode of reproduction in combination with the primarily boleochorous mode of dispersal leading to spatial clustering of individuals belonging to highly differentiated familial lineages.

### 4.2 Differences in genetic diversity levels between river and anthropogenic habitats

Near‐natural river habitats showed significantly higher levels of genetic diversity than the two anthropogenic pond types. Secondary habitats must therefore be regarded as genetically impoverished. Our data suggest that the proportion of selfing (as approximated by the inbreeding coefficient when population sizes are rather large; Figure 2) as well as the dynamics between the soil seed bank and the above‐ground population are intrinsic characteristics of *Cyperus fuscus*, which do not depend on habitat type. Drift, altered patterns of dispersal and differences in the strength of selection pressures could be responsible for the unequal levels of genetic diversity.

It is probable that some founder effect occurred when *Cyperus fuscus* first colonized sites provided by fish farming, which was initiated more than 600 years ago in our study region (Květ et al., 2002). The first documented records of *Cyperus fuscus* in fish farms in the Czech Republic date back to the first half of the 19th century (Kaplan et al., 2016). *Cyperus fuscus* prefers mineral‐rich basic soils, which can be found in northern Bohemia (where populations F1 and S1‒3 have been sampled). In contrast, the fishpond regions in western and southern Bohemia (populations F2‒9 and S4‒10) and south‐western Moravia (population F10) are mainly formed by mineral‐poor acidic soils. Consequently, *Cyperus fuscus* was rare in most parts of southern Bohemia until the 1950s, where it occurred mainly in eutrophic water bodies in settlements (Hejný, 1960). Because of overall eutrophication and soil chemistry changes associated with fish farming intensification, the number of records considerably increased since then (Kaplan et al., 2016; Šumberová, 2003). Populations F6–9 and S6‒8, all from nearby localities in the western South Bohemian and eastern Plzeň Regions, differ from the remaining pond populations in being genetically virtually identical (Figure 4). It is difficult to know what caused this genetic identity, because the history of construction and management of the localities is largely unknown. A population expansion after a bottleneck, which might have been caused by changes in management and land use, is one possible explanation for the genetic identity. In the year 2012, above‐ground population sizes were larger on average in the anthropogenic pond habitats than in river habitats (Appendix S1), so that there is no reason to assume genetic drift because of small population sizes any more.

The analysis of recent migration also helps to explain the maintenance of different genetic diversity levels based on altered levels and patterns of gene flow. Our data suggest that high rates of immigration enrich the gene pool that may have been impoverished through drift. River and secondary habitats show fundamental differences in regional dynamics and dispersal. It is visible that populations along rivers exchange many migrants in every generation (Figure5). Less dispersal is detectable in secondary habitats. However, effective seed dispersal is essential for the maintenance of genetic diversity for annual selfing species, which cannot benefit from an outcrossing mating system (Yan et al., 2009). Bidirectional ornithochory (e.g. Deil, 2005) or ichthyochory seems to contribute more to dispersal along river corridors than unidirectional hydrochory (Nilsson, Brown, Jansson, & Merritt, 2010), as immigrants are almost equally likely to stem from populations upstream or downstream along the river systems Danube, Elbe, and Oder. The dynamics in river habitats has also been examined in the annual *Erysimum cheiranthoides* growing on stony riverbanks along the river Meuse (Honnay et al., 2009). The species behaves as a very dynamic metapopulation and, similarly to our study, the metapopulation showed evidence of extensive and bidirectional dispersal, downstream and upstream along the river corridor. In a literature survey, Wubs et al. (2016) found that upstream dispersal over tens and sometimes hundreds of kilometers was detected in the majority of examined stream and riparian plant species. Waterfowl that use rivers as a migration corridor are regular and efficient seed dispersers of wetland plants. Viable seeds of many common wetland species can be transported by ducks on feathers and crusts of mud as well as in their guts over long distances (several hundreds of kilometers), whereby small seeds have the highest potential to be dispersed (Bílý, Mourková, & Bergmann, 2008; Figuerola & Green, 2002; Hohensee & Frey, 2001; Kleyheeg, Leeuwen, Morison, Nolet, & Soons, 2015; Soons et al., 2008; Viana, Santamaría, & Figuerola, 2016). *Cyperus fuscus* produces an enormous amount of small nutlets (Bryson & Carter, 2010), so that ducks should be ideal dispersers of this species. Although we do not yet have direct evidence of the dispersal of *Cyperus fuscus* by waterfowl, our own field observations suggest that this species is a common part of the mallard, but also swan and goose diet. In contrast to fish storage ponds, fishponds are attractive for waterfowl, also for breeding (Bílý et al., 2008; Kameníková & Rajchard, 2013), but the rate of immigration from rivers is nevertheless greatly reduced in fishponds (Figure5). If the birds are breeding, they occur in one and the same fishpond, later in a relatively small area of several fishponds. In the fishponds, the plants usually germinate earlier than in the rivers. Thus, in the time of autumn migration, the plants in fishponds are usually dead (not attractive for grazing birds) or the exposed fishpond bottoms are even completely flooded. Ichthyochory may contribute to seed dispersal when the fish caught in fishponds is stocked in fish storage ponds. We suggest that in the secondary habitats, anthropochory becomes increasingly important. Šumberová, Ducháček, et al. (2012) demonstrated that seeds of wetland ephemerals are dispersed between fishponds and fish storage ponds with mud on cars, rubber boots, and tools of the fish farmers. Anthropochorous dispersal should mainly be on short distances between ponds of a fish farming company and so should not substantially increase genetic diversity.

### 4.3 Only moderate genetic differentiation among habitat types despite differential adaptation

Strong selection pressures in anthropogenic habitat types could serve as another explanation for the observed reduced genetic diversity levels. Because *Cyperus fuscus* is highly selfing, selective sweeps and/or background selection against deleterious alleles should be able to reduce genetic diversity in large parts of the genome (Roze, 2016). It has been hypothesized that novel habitats created by humans may provide evolutionary opportunities for rapid adaptive niche shifts associated with lineage divergence (e.g., Baduel, Hunter, Yeola, & Bomblies, 2018; Kamdem et al., 2012). Previously, we found evidence for phenotypic differentiation between primary and secondary habitats in our study species suggesting that adaptive evolution has taken place (Böckelmann et al., 2017). Plants from river habitats tolerated flooding events that usually occur unpredictably regarding particular date and intensity in early summer on riverbanks better than plants from the anthropogenic pond types. On the other extreme, plants from fish storage ponds followed a “develop fast, but stay dwarf” strategy, which seemingly allows them to cope with drought stress and numerous and irregular disturbances. Similarly, such a rapid cycling strategy has also been demonstrated for ruderals such as *Arabidopsis arenosa* populations along railways, which are affected by truncation selection through drought and/or extermination of plants as a management measure (Baduel et al., 2018). In contrast to *Arabidopsis arenosa*, where railway populations are clearly genetically distinct from populations found in other habitats (Arnold, Kim, & Bomblies, 2015), however, genetic differentiation among habitat types was in the same range as among biogeographical regions or river systems in *Cyperus fuscus* (Table3). Possible reasons for the only moderate differentiation include the use of putatively neutral markers (e.g., Hodel et al., 2016; to trace adaptive evolution is problematic for that reason). Alternatively, it may suggest that random genetic backgrounds have been picked up during adaptation processes occurring in parallel in local populations, that various alleles/loci may confer the phenotypes observed in the anthropogenic pond habitats (Baduel et al., 2018), and/or that the differences have an epigenetic, rather than a genetic basis (Bossdorf et al., 2008). High gene flow may not interfere with local adaptation because gene flow may be “assortative” in the sense that it normally occurs between sites with similar selection regime. For instance, dispersal by waterfowl should connect sites along river corridors, whereas unintentional anthropogenic dispersal by fish farming activities should connect various pond types.

### 4.4 High levels of gene flow and a south‐north as well as east‐west gradient in private allelic richness across Europe

The star‐like Neighbor‐Net network (Figure4) shows the typically high degree of differentiation among individual (even nearby) populations in selfing species (Koornneef, Alonso‐Blanco, & Vreugdenhil, 2004; Nordborg et al., 2005) and weak association between geographic and genetic distance across Europe. While migrant analysis testifies to the recent dispersal of individuals, analysis of genetic isolation by distance sums up historical gene flow over many generations. Both analyses suggest that *Cyperus fuscus* is a highly mobile species. The Mantel correlogram indicates genetic resemblance of populations up to ~285 km distant across the European scale.

We found the highest private allelic richness in the Mediterranean region and a decreasing east‐to‐west gradient in private allelic richness in temperate Europe, from the Pannonian to the Continental to the Atlantic region (Figures3, 7; Appendix S5). European southern glacial refugia have been postulated in the Iberian Peninsula, Italy, the Balkans, Anatolia, and the region around the Black Sea for a range of plant and animal species (Ali et al., 2017; Bartish, Kadereit, & Comes, 2006; Comes & Kadereit, 1998; Hewitt, 1996). The occurrence of private alleles suggests a long‐lasting in situ history (Médail & Diadema, 2009). Private alleles might then have been lost through drift during the supposed postglacial migration. Our results suggest a postglacial colonization of temperate Europe from southern and eastern refugia (the Mediterranean and eventually the region around the Black Sea, from where no samples were available in this study). The lack of a strong phylogeographic structure can be explained by efficient dispersal and a rapid postglacial expansion.

High values of allelic richness and expected heterozygosity in Pannonian populations may relate to the drier and warmer present Pannonian climate (when compared to the Continental and Atlantic climate). Due to frequent water‐level fluctuations in dry and warm regions, suitable habitats with exposed fine muddy sediments may appear with higher frequency in the landscape. In the present Mediterranean climate, on the other hand, quickly desiccating exposed substrata‐like sand or gravel may not support similarly large populations of *Cyperus fuscus*. Long‐term observation of populations in the various regions and a better population sampling for estimation of genetic diversity in the Mediterranean region would be needed to evaluate this hypothesis.

### 4.5 Implications for conservation

Freshwater wetland habitats and particularly riverine floodplains face a dramatic decline (Hein et al., 2016; Tockner & Stanford, 2002). In a widely anthropogenically influenced and fragmented Central Europe, the role of man‐made wetland habitats possibly harboring rare and endangered species should not be neglected (Deil, 2005; Květ et al., 2002; Richert et al., 2016). Because populations in river alluvia diminished, *Cyperus fuscus* is classified as vulnerable in Austria, the Czech Republic, Great Britain, Liechtenstein, and Switzerland (Bornand et al., 2016; Broggi, Waldburger, & Staub, 2006; Cheffings & Farrell, 2005; Grulich, 2012; Niklfeld & Schratt‐Ehrendorfer, 1999), but it still thrives in secondary habitats. A previous study suggested that populations from river habitats are fitter overall and better adapted to repeated and severe flooding (Böckelmann et al., 2017). The present study suggests that populations from secondary habitats suffer from reduced genetic diversity levels. Thus, our case study illustrates the need of conserving the dynamic metapopulations along river systems with frequent destruction and new generation of habitat patches by the activity of the river and frequent dispersal, if one wants to conserve all of the genetic diversity (and hence adaptive potential) in the species and, more generally, in the water–land interface vegetation of wetland habitats (Burkart, 2001). This highlights the importance of protecting naturally flowing rivers and restoring morphology and hydrological dynamics of particularly those regulated rivers that still retain some level of ecological integrity (Tockner & Stanford, 2002).

## CONFLICT OF INTEREST

The authors declare no conflict of interest.

## AUTHORS’ CONTRIBUTION

K.G.B., H.G., K.Š., and K.T. designed the experiment; J.B. and G.K. acquired the data; J.B., K.G.B., H.G., K.Š., and K.T. analyzed and interpreted the data; J.B., K.Š., and K.T. drafted the article, and all other coauthors critically revised and approved it.

## Supporting information

Appendix S1‐S5

## Data Availability

Sampling locations and microsatellite genotypes: Dryad https://doi.org/10.5061/dryad.05qfttdzv
