# Supplementary material for: Genetic variation in an ephemeral mudflat species: The role of the soil seed bank and dispersal in river and secondary anthropogenic habitats
Source: Ecol Evol. 2020 Mar 17;10(8):3620–35. doi: 10.1002/ece3.6109 (PMC7160169; doi:10.1002/ece3.6109)
Supplement: Supplementary file 1 — Appendix S1‐S5 [file ECE3-10-3620-s001.pdf]

Appendix S1. Populations used for analysis. Collectors: AM, A. Mesterházy, CB, C. Berg, ENB, E. Naumer-Bernhardt, EW, E. Weeda, FD, F. Dunkel, FL, F. Landucci, FP, F. Pinet, HG, H. Gasté, HR, H. Ringel, HS, H. Sluschny, IV, I. Varga, JB, J. Böckelmann, KB, K. Bubíková, KGB, K.-G. Bernhardt, KŠ, K. Šumberová, KT, K. Tremetsberger, KW, K. Wernisch, MK, M. Kropf, MW, M. Wernisch, NS, N. Stöckl, PK, P. Kúr, RG, R. Guarino, RL, R. Lansdown, SP, S. Píšová, SW, S. Wittwer, UR, U. Raabe, ZH, Z. Hroudová, ZK, Z. Kącki. Index Herbariorum (http://sweetgum.nybg.org/science/ih/) code: WHB, herbarium of the University of Natural Resources and Life Sciences, Vienna.

Population code	Locality	Habitat	Geographic coordinates (altitude)	Collector(s) (coll. date)	Voucher number	Estimated above-ground population size
**Populations belonging to the 'river' habitat in Central Europe (altitudinal range = 51–182 m)**						
R1	Poland, Lower Silesia, Cigacice	Artificial side pool of river Oder, exposed river bank	52.03138°N, 15.61098°E (51 m)	JB, KŠ, PK, ZK (3.10.2012)	WHB 62955	10^4 individuals
R2	Poland, Lower Silesia, Borków	Branch of river Oder	51.67462°N, 16.20398°E (75 m)	JB, KŠ, PK, ZK (2.10.2012)	WHB 62973	2.5×10^4 individuals
R3	Czech Republic, Ústí nad Labem Region, Nebočady	Artificial side pool of river Elbe	50.72945°N, 14.18703°E (127 m)	JB, KŠ, PK, SP (13.9.2012)	WHB 62978	2,000 individuals
R4	Czech Republic, Central Bohemian Region, Záryby	Shoreline of river Elbe, partly disturbed	50.22383°N, 14.62862°E (165 m)	JB, KŠ, KT, PK, SP, ZH (21.8.2012)	WHB 62957	1,500 individuals
R5	Czech Republic, South Moravian Region, Velké Němčice	Alluvial sediments of river Svratka	48.98427°N, 16.66490°E (179 m)	JB, KŠ, KT (11.9.2012)	WHB 62979	500 individuals
R6	Czech Republic, South Moravian Region, Moravská Nová Ves	Oxbow of river Morava	48.78465°N, 17.07988°E (159 m)	JB, KŠ, KT (11.9.2012)	WHB 62980	2.5×10^4 individuals
R7	Czech Republic, South Moravian Region, Lanžhot	Shoreline of river Thaya	48.71183°N, 16.90282°E (153 m)	JB, KŠ, KT (10.9.2012)	WHB 62982	1,500 individuals

Appendix S1. Continued.

Population code	Locality	Habitat	Geographic coordinates (altitude)	Collector(s) (coll. date)	Voucher number	Estimated above-ground population size
R8	Czech Republic, South Moravian Region, Lanžhot	Branch of river Thaya	48.67257°N, 16.92403°E (153 m)	JB, KŠ, KT (10.9.2012)	WHB 62981	5,000 individuals
R9	Slovakia, Bratislava Region, Vysoká pri Morave	Oxbow of river Morava	48.31232°N, 16.90373°E (148 m)	JB, KŠ, PK (5.10.2012)	WHB 62972	5,000 individuals
R10	Austria, Lower Austria, Markthof	Branch of river Morava, alluvial sediments	48.19142°N, 16.97170°E (144 m)	JB, KŠ, PK (5.10.2012)	WHB 62974	500 individuals
R11	Austria, Lower Austria, Zwentendorf	Shoreline of river Traisen	48.37075°N, 15.83806°E (182 m)	JB (18.9.2012)	WHB 62956	300 individuals
**Populations belonging to the 'fishpond' habitat in Central Europe (altitudinal range = 251–469 m)**						
F1	Czech Republic, Liberec Region, Zahrádky	Fishpond Novozámecký, used for marketable fish, dried after many years due to dam reconstruction	50.62812°N, 14.54325°E (251 m)	JB, KŠ, KT (10.7.2012)	WHB 62967, 62968	2.5×10^4 individuals
F2	Czech Republic, Central Bohemian Region, Petrovice	Fishpond Horní Petrovický, used for fish fry, regularly dried in summer	49.71832°N, 14.65050°E (404 m)	JB, KŠ, KT (11.7.2012)	WHB 62965, 62966	5,000 individuals
F3	Czech Republic, Central Bohemian Region, Libohošť	Fishpond Libohošťský, used for fish fry, regularly dried in summer	49.70095°N, 14.58997°E (376 m)	JB, KŠ, KT (11.7.2012)	WHB 62964	5,000 individuals
F4	Czech Republic, Central Bohemian Region, Sedlečko	Fishpond Velký Sedlečský, used for marketable fish, in biennial intervals with low water level	49.69357°N, 14.53485°E (445 m)	JB, KB (27.7.2012)	WHB 62994	4×10^4 individuals

Appendix S1. Continued.

Population code	Locality	Habitat	Geographic coordinates (altitude)	Collector(s) (coll. date)	Voucher number	Estimated above-ground population size
F5	Czech Republic, Central Bohemian Region, Solopysky	Fishpond Dolní Solopyský, management unknown, very often low water level	49.65362°N, 14.38492°E (381 m)	JB, KB (2.8.2012)	WHB 62992	3,000 individuals
F6	Czech Republic, South Bohemian Region, Skaličany	Fishpond Pýcha, used for fish fry, regularly dried in summer	49.43463°N, 13.91165°E (442 m)	JB, KB (24.7.2012)	WHB 62996	2.5×10^4 individuals
F7	Czech Republic, Plzeň Region, Smrkovec	Fishpond Velký Smrkovec, used for marketable fish, regularly with low water level due to small inflow	49.33685°N, 13.59858°E (469 m)	JB, KB (24.7.2012)	WHB 62997	2,000 individuals
F8	Czech Republic, South Bohemian Region, Rojice	Fishpond Velkorojický, used for marketable fish, low water level after about 5 years	49.34997°N, 13.94233°E (457 m)	JB, KŠ, KT (13.7.2012)	WHB 62961	2.5×10^4 individuals
F9	Czech Republic, South Bohemian Region, Chrášťovice	Fishpond Chválovec, used for fish fry, regularly dried in summer	49.32103°N, 13.89727°E (466 m)	JB, KŠ, KT (13.7.2012)	WHB 62962	5,000 individuals
F10	Czech Republic, South Moravian Region, Křepice	Unnamed small village fishpond, drained after many years due to dam reconstruction	48.98655°N, 16.09402°E (335 m)	KB (5.9.2012)	WHB 62983	3,000 individuals
**Populations belonging to the 'fish storage pond' habitat in Central Europe (altitudinal range = 251–453 m)**						
S1	Czech Republic, Liberec Region, Doksy	Fish storage ponds with long summer drainage and grazing	50.56383°N, 14.65887°E (276 m)	JB, KŠ, KT (10.7.2012)	WHB 62969	5,000 individuals
S2	Czech Republic, Central Bohemian Region, Střehom	Fish storage ponds with short summer drainage, mowing and herbicide spraying	50.47235°N, 15.13253°E (251 m)	JB, KŠ, KT, SP (23.8.2012)	WHB 62985	4,000 individuals

Appendix S1. Continued.

Population code	Locality	Habitat	Geographic coordinates (altitude)	Collector(s) (coll. date)	Voucher number	Estimated above-ground population size
S3	Czech Republic, Central Bohemian Region, Mšec	Fish storage ponds with long summer drainage and herbicide spraying	50.19692°N, 13.91085°E (407 m)	JB, KŠ, KT (12.7.2012)	WHB 62963	5,000 individuals
S4	Czech Republic, Central Bohemian Region, Semovice	Fish storage pond with short summer drainage and occasional herbicide spraying	49.75112°N, 14.66092°E (354 m)	JB, KB (6.8.2012)	WHB 62959	5,000 individuals
S5	Czech Republic, Central Bohemian Region, Dobrá Voda	Fish storage pond with long summer drainage and sheep grazing	49.55412°N, 13.99650°E (448 m)	JB, KB (9.8.2012)	WHB 62988	4,000 individuals
S6	Czech Republic, South Bohemian Region, Tchořovice	Fish storage pond with long summer drainage and mowing	49.43525°N, 13.80737°E (453 m)	JB, KB (9.8.2012)	WHB 62987	3,000 individuals
S7	Czech Republic, South Bohemian Region, Rojice	Fish storage pond with long summer drainage, mowing and low-intensity poultry grazing	49.34737°N, 13.94838°E (452 m)	JB, KB, KŠ (8.8.2012)	WHB 62989	5,000 individuals
S8	Czech Republic, South Bohemian Region, Čejetice	Fish storage pond with long summer drainage and mowing	49.24955°N, 14.02218°E (382 m)	JB, KB, KŠ (8.8.2012)	WHB 62958	500 individuals
S9	Czech Republic, South Bohemian Region, Hluboká nad Vltavou	Fish storage pond with long summer drainage and herbicide spraying	49.04477°N, 14.43318°E (372 m)	JB, KB, KŠ (7.8.2012)	WHB 62991	3,000 individuals
S10	Czech Republic, South Bohemian Region, Hluboká nad Vltavou	Fish storage pond with short summer drainage and herbicide spraying	49.04373°N, 14.43253°E (374 m)	JB, KŠ, KT (14.7.2012)	WHB 62960	5,000 individuals

Appendix S1. Continued.

Population code (no. of ind. analysed)	Locality and habitat	Geographic coordinates (altitude)	Collector(s) (collection date)	Voucher number
**Additional populations from the Mediterranean region (altitudinal range = 1–313 m)**				
ES1 (12)	Spain, Tarragona Province, Tortosa, muddy depression and shore of river Ebro	40.81653°N, 0.52152°E (6 m)	KGB (16.9.2004)	WHB 45881, 45882
ES2 (11)	Spain, Valencia Province, Segorbe, muddy shore of river Palancia	39.857°N, 0.489°W (313 m)	KGB (19.9.2004)	WHB 45880
FR1 (11)	France, Corsica, camping site U Sortipiani, gravel bank of river Tavignano	42.21076°N, 9.31501°E (146 m)	KT (17.8.2012)	WHB 71953–71959
GR1 (1)	Greece, Peloponnese Region, rice field S road Kalamata – Messini near airport	37.04861°N, 22.03056°E (1 m)	UR (9.10.2013)	WHB 61363, 61364
GR2 (3)	Greece, Peloponnese Region, along road Vrontamas – Lefkochoma at bridge over river Eurotas, river bank	36.97361°N, 22.58056°E (95 m)	UR (18.10.2013)	WHB 61361, 61362
HR1 (12)	Croatia, Istria County, river Mirna, at bridge to Motovun	45.34561°N, 13.82958°E (14 m)	KGB, ENB (29.8.2013)	WHB 59431, 59432, 59477, 59478
HR2 (18)	Croatia, Istria County, Krk island, N Krk city, along dirt road, pond, muddy shore almost free of vegetation	45.06194°N, 14.59000°E (100 m)	KGB (29.9.2009)	WHB 50374, 50375
IT1 (9)	Italy, Lombardy Region, at river Ticino, Parco Ticino, river gravel, with moist, sandy depressions	45.23600°N, 9.00642°E (63 m)	KGB, ENB (31.8.2013)	WHB 59476, 60143, 60144
IT2 (2)	Italy, Lombardy Region, NE San Biagio, rice field, wet	45.21367°N, 8.98283°E (71 m)	KGB, ENB (31.8.2013)	WHB 60174
IT3 (12)	Italy, Umbria Region, San Savino, Lago Trasimeno, artificial channel tributary of the lake, exceptionally dry for a long time in the year of collection	43.10590°N, 12.18650°E (258 m)	FL (19.9.2012)	WHB 60744–60748
IT4 (11)	Italy, Sicily Region, Palermo, mouth of river Oreto, at and below the bridge (Via Messina Marine), alluvial sediments on concrete, moist	38.10996°N, 13.37978°E (1 m)	KGB (13.9.2013)	WHB 59535, 59536, 59550, 59551

Appendix S1. Continued.

Population code (no. of ind. analysed)	Locality and habitat	Geographic coordinates (altitude)	Collector(s) (collection date)	Voucher number
IT5 (10)	Italy, Sicily Region, along road Segesta – Castellammare del Golfo, Abbeveratoio Pioppa, source with enclosed basin and overflow	37.98658°N, 12.90039°E (130 m)	KGB, RG (11.9.2013)	WHB 59537–59539
TR (1)	Turkey, Marmara Region, Yeniköy SW Trojan Ruins, near the beach	39.92097°N, 26.15691°E (3 m)	MK (30.6.2013)	WHB 59717
**Additional populations from the Pannonian region in Europe (altitudinal range = 78–211 m)**				
AT1 (12)	Austria, Burgenland, c. 1.5 km ENE Zurndorf, fallow field, partly moist	47.98919°N, 17.03906°E (131 m)	KGB, NS, KT (7.8.2013)	WHB 60778
AT2 (17)	Austria, Burgenland, E Zurndorf, oxbow of river Leitha, sandy mud	47.97319°N, 17.05294°E (131 m)	KGB (25.9.2013)	WHB 60491–60494
AT3 (20)	Austria, Burgenland, E Zurndorf, dirt road to Zurndorf, field next to river Leitha	47.97250°N, 17.05308°E (130 m)	KGB (17.7.2013)	WHB 59713–59716
AT4 (10)	Austria, Burgenland, c. 1 km N Nickelsdorf, fallow field next to river Leitha, partly moist	47.96361°N, 17.06914°E (129 m)	KGB, NS, KT (7.8.2013)	WHB 60779
HR3 (9)	Croatia, Osijek-Baranja County, NE Osijek, S Podunavlje, at bridge over channel, mud bank	45.62588°N, 18.81360°E (78 m)	MW, KW (15.10.2011)	WHB 54394, 54395
HU1 (6)	Hungary, Vas County, Győrvár, edge of pond	46.97967°N, 16.83164°E (153 m)	AM (14.10.2012)	WHB 60760–60762
HU2 (7)	Hungary, Komárom-Esztergom County, Ács, open bank of rivulet	47.74097°N, 18.00204°E (110 m)	AM (16.10.2012)	WHB 60768
HU3 (12)	Hungary, Győr-Moson-Sopron County, Győr, in wet, open disturbed sand	47.64374°N, 17.60800°E (112 m)	AM (17.10.2012)	WHB 60751, 60752
HU4 (8)	Hungary, Veszprém County, Pápa-Nóráp, shore of reservoir	47.27789°N, 17.46288°E (146 m)	AM (17.10.2012)	WHB 60750
HU5 (9)	Hungary, Veszprém County, Ajka-Tósokberénd, lake shore	47.09707°N, 17.52416°E (211 m)	AM (14.10.2012)	WHB 60757, 60758

Appendix S1. Continued.

Population code (no. of ind. analysed)	Locality and habitat	Geographic coordinates (altitude)	Collector(s) (collection date)	Voucher number
HU6 (2)	Hungary, Zala County, Gyenesdiás, bottom of canal near lake Balaton	46.76437°N, 17.27009°E (104 m)	AM (8.9.2012)	WHB 60763
HU7 (7)	Hungary, Somogy County, Varászló, fish storage pond	46.44686°N, 17.19489°E (125 m)	AM (11.10.2012)	WHB 60759
HU8 (11)	Hungary, Fejér County, Dinnyés, fishpond	47.18046°N, 18.53974°E (103 m)	AM (12.9.2012)	WHB 60741–60743
HU9 (4)	Hungary, Pest County, Szentendre, river banks of river Danube	47.66503°N, 19.07951°E (101 m)	AM (11.9.2012)	WHB 60749
HU10 (8)	Hungary, Heves County, Egerszalók, side of ditch	47.85389°N, 20.32988°E (150 m)	AM (6.10.2012)	WHB 60765, 60766
HU11 (11)	Hungary, Jász-Nagykun-Szolnok County, Tiszafüred, Tisza wetlands	47.60865°N, 20.70626°E (85 m)	MK (14.7.2009)	WHB 59514
HU12 (7)	Hungary, Szabolcs-Szatmár-Bereg County, Tiszabecs, open bank of river Tisza	48.10706°N, 22.83113°E (112 m)	IV (10.10.2012)	WHB 60767
SK1 (10)	Slovakia, Trnava Region, NNW Tomky, fishpond Vodná Nádrž Horná Studená Voda, sandy pond bank	48.58410°N, 17.07905°E (168 m)	KT, PK (20.9.2013)	WHB 60756
SK2 (8)	Slovakia, Bratislava Region, SW Malé Levaré, Autocamping Rudava, gravelly pond bank	48.49391°N, 16.96058°E (147 m)	KT, PK (19.9.2013)	WHB 60754
SK3 (10)	Slovakia, Bratislava Region, Senec, leisure facility Slečné Jazerá, gravelly pond bank	48.21523°N, 17.41502°E (121 m)	KT, PK (20.9.2013)	WHB 60755
SK4 (9)	Slovakia, Bratislava Region, pond Kuchajda near station Nové Mesto (Bratislava), gravelly pond bank	48.17098°N, 17.14310°E (133 m)	KT, PK (19.9.2013)	WHB 60753
**Additional populations from the Continental region in Europe (altitudinal range = 11–475 m)**				
AT5 (9)	Austria, Steiermark, Graz, botanical garden, pond	47.08217°N, 15.45632°E (377 m)	CB (unknown)	No voucher
CH (12)	Switzerland, St. Gallen, Saxer Riet, grassland renaturation, pond bank	47.22243°N, 9.47773°E (438 m)	KGB (8.9.2004)	WHB 45887, 45888

Appendix S1. Continued.

Population code (no. of ind. analysed)	Locality and habitat	Geographic coordinates (altitude)	Collector(s) (collection date)	Voucher number
DE1 (10)	Germany, Mecklenburg-Vorpommern, NW Feldberg, Sprockfitz	53.35251°N, 13.40753°E (89 m)	HR (20.7.2013)	No voucher
DE2 (11)	Germany, Mecklenburg-Vorpommern, muddy shoreline of river Elbe between two spur dikes c. 1.65 km ESE Rüterberg, stand of *Bidens radiata*	53.14505°N, 11.20748°E (11 m)	HS (2013)	No voucher
DE3 (7)	Germany, Sachsen-Anhalt, Bleddin, oxbow of river Elbe	51.78579°N, 12.79118°E (72 m)	FD, SW (13.9.2014)	Voucher in collection F. Dunkel
DE4 (9)	Germany, Baden-Württemberg, gravel pit Meißenheim	48.43082°N, 7.76324°E (147 m)	PK (20.7.2013)	WHB 71951, 71952
LI1 (12)	Liechtenstein, Schaan, Unterau, nursery garden near river Rhine	47.18722°N, 9.49222°E (446 m)	KGB (8.9.2004)	WHB 45889, 45890
LI2 (12)	Liechtenstein, Schaan, Unterau, newly established small water body	47.18250°N, 9.48889°E (454 m)	KGB (8.9.2004)	WHB 45883
LI3 (12)	Liechtenstein, between Mäls and Balzers, newly established pond, moist depressions	47.06335°N, 9.50167°E (475 m)	KGB (8.9.2004)	WHB 45884–45886
LT (15)	Lithuania, Vilnius County, Vilnius, at hotel Park Villa, sandy river bank of river Neris	54.73125°N, 25.30064°E (95 m)	KGB (12.9.2014)	WHB 62047, 62048
**Additional populations from the Atlantic region in Europe (altitudinal range = 4–113 m)**				
FR2 (10)	France, Centre-Val de Loire Region, W Saulnay, unnamed pond	46.86301°N, 1.25556°E (113 m)	FP, HG (10.10.2014)	No voucher
FR3 (9)	France, Centre-Val de Loire Region, N Rosnay, pond Piat	46.73191°N, 1.20580°E (98 m)	FP, HG (30.9.2014)	No voucher
GB1 (1)	United Kingdom, England, South West Region, Walton-in-Gordano, Gordano Valley	51.44971°N, 2.81628°W (7 m)	RL (5.9.2013)	No voucher
GB2 (10)	United Kingdom, England, South East Region, Breamore Marsh	50.96302°N, 1.77899°W (33 m)	RL (4.9.2013)	No voucher

Appendix S1. Continued.

Population code (no. of ind. analysed)	Locality and habitat	Geographic coordinates (altitude)	Collector(s) (collection date)	Voucher number
NL (10)	Netherlands, Overijssel Province, Zwolle, depression in bank of river IJssel	52.4969°N, 6.0599°E (4 m)	EW (5.7.2014)	WHB 61387, 61388

Appendix S2. Measures of genetic diversity (mean ± standard error over loci) based on 21 microsatellite markers of populations in Central Europe (grouped by habitat), each population differentiated in the above-ground (AG) and soil seed bank (S: S_s, shallow seed bank; S_d, deep seed bank) fractions, respectively. *N*, number of individuals analysed; A_r, allelic richness; pA_r, private allelic richness; H_I, average observed heterozygosity; H_S, average expected heterozygosity under Hardy-Weinberg equilibrium; and F_{IS}, inbreeding coefficient. A_r and pA_r were determined based on 10 randomly sampled alleles per fraction and population; pA_r was determined by pairwise comparison of the above-ground and soil fractions in each population separately. See Appendix S1 for a list of population codes with collection information.

Population	Biogeogr. region River system	Fraction	*N*	A_r	pA_r	H_I	H_S	F_{IS}
**Rivers**								
R1	Continental	AG	15	2.46 ± 0.18	0.86 ± 0.18	0.20 ± 0.02	0.42 ± 0.05	0.52 ± 0.04
	Oder	S (S_s, S_d)	6 (0, 6)	1.69 ± 0.14	0.08 ± 0.04	0.12 ± 0.03	0.25 ± 0.05	0.39 ± 0.09
R2	Continental	AG	17	2.54 ± 0.15	0.41 ± 0.08	0.35 ± 0.03	0.47 ± 0.04	0.25 ± 0.05
	Oder	S (S_s, S_d)	15 (5, 10)	2.53 ± 0.13	0.40 ± 0.07	0.20 ± 0.02	0.47 ± 0.03	0.56 ± 0.05
R3	Continental	AG	15	2.69 ± 0.16	0.64 ± 0.16	0.18 ± 0.02	0.48 ± 0.04	0.62 ± 0.05
	Elbe	S (S_s, S_d)	5 (0, 5)	2.19 ± 0.15	0.14 ± 0.04	0.01 ± 0.01	0.44 ± 0.04	0.98 ± 0.01
R4	Continental	AG	15	2.20 ± 0.14	0.61 ± 0.10	0.04 ± 0.01	0.35 ± 0.04	0.88 ± 0.03
	Elbe	S (S_s, S_d)	8 (2, 6)	1.90 ± 0.17	0.31 ± 0.10	0.01 ± 0.01	0.25 ± 0.05	0.96 ± 0.02
R5	Pannonian	AG	15	2.79 ± 0.19	0.77 ± 0.14	0.05 ± 0.01	0.47 ± 0.04	0.89 ± 0.03
	Danube	S (S_s, S_d)	15 (5, 10)	2.49 ± 0.16	0.47 ± 0.09	0.09 ± 0.01	0.47 ± 0.04	0.77 ± 0.05
R6	Pannonian	AG	24	3.10 ± 0.18	0.72 ± 0.14	0.29 ± 0.02	0.58 ± 0.03	0.49 ± 0.03
	Danube	S (S_s, S_d)	14 (0, 14)	2.95 ± 0.20	0.57 ± 0.12	0.21 ± 0.03	0.54 ± 0.04	0.60 ± 0.04
R7	Pannonian	AG	16	2.93 ± 0.16	0.93 ± 0.20	0.08 ± 0.01	0.54 ± 0.03	0.86 ± 0.02
	Danube	S (S_s, S_d)	5 (0, 5)	2.57 ± 0.16	0.57 ± 0.16	0.14 ± 0.03	0.45 ± 0.04	0.63 ± 0.08
R8	Pannonian	AG	15	2.99 ± 0.21	0.58 ± 0.14	0.13 ± 0.02	0.58 ± 0.03	0.77 ± 0.03
	Danube	S (S_s, S_d)	20 (5, 15)	2.85 ± 0.20	0.44 ± 0.13	0.15 ± 0.02	0.56 ± 0.03	0.74 ± 0.03
R9	Pannonian	AG	13	2.58 ± 0.14	0.54 ± 0.12	0.14 ± 0.02	0.44 ± 0.03	0.65 ± 0.06
	Danube	S (S_s, S_d)	8 (1, 7)	2.57 ± 0.17	0.52 ± 0.13	0.20 ± 0.03	0.48 ± 0.04	0.55 ± 0.05
R10	Pannonian	AG	14	3.16 ± 0.17	0.78 ± 0.12	0.06 ± 0.01	0.57 ± 0.03	0.89 ± 0.03
	Danube	S (S_s, S_d)	8 (3, 5)	2.92 ± 0.24	0.55 ± 0.15	0.21 ± 0.03	0.55 ± 0.04	0.59 ± 0.05

Appendix S2. Continued.

Population	Biogeogr. region River system	Fraction	N	A_r	pA_r	H_I	H_S	F_{IS}
R11	Pannonian	AG	15	1.83 ± 0.11	0.11 ± 0.05	0.07 ± 0.02	0.26 ± 0.03	0.74 ± 0.05
	Danube	S (S_s, S_d)	17 (2, 15)	2.04 ± 0.12	0.32 ± 0.08	0.04 ± 0.01	0.42 ± 0.04	0.90 ± 0.02
Mean ± standard deviation		AG	16	2.66 ± 0.40	0.63 ± 0.23	0.15 ± 0.10	0.47 ± 0.10	0.69 ± 0.20
		S (S_s, S_d)	11 (2, 9)	2.43 ± 0.42	0.40 ± 0.17	0.13 ± 0.08	0.44 ± 0.11	0.70 ± 0.19
**Fishponds**								
F1	Continental	AG	22	2.07 ± 0.13	0.33 ± 0.09	0.14 ± 0.02	0.38 ± 0.04	0.63 ± 0.04
	Elbe	S (S_s, S_d)	26 (4, 22)	1.94 ± 0.08	0.19 ± 0.04	0.09 ± 0.01	0.34 ± 0.04	0.74 ± 0.05
F2	Continental	AG	22	1.22 ± 0.07	0.09 ± 0.03	0.01 ± 0.01	0.06 ± 0.03	0.91 ± 0.03
	Elbe	S (S_s, S_d)	34 (10, 24)	1.34 ± 0.07	0.21 ± 0.05	0.00 ± 0.00	0.09 ± 0.02	0.98 ± 0.01
F3	Continental	AG	20	2.08 ± 0.11	0.14 ± 0.04	0.05 ± 0.01	0.41 ± 0.04	0.86 ± 0.03
	Elbe	S (S_s, S_d)	21 (2, 19)	2.28 ± 0.11	0.33 ± 0.07	0.17 ± 0.02	0.45 ± 0.03	0.60 ± 0.05
F4	Continental	AG	16	1.13 ± 0.05	0.13 ± 0.05	0.00 ± 0.00	0.03 ± 0.01	1.00 ± 0.00
	Elbe	S (S_s, S_d)	9 (0, 9)	1.00 ± 0.00	0.00 ± 0.00	0.00 ± 0.00	0.00 ± 0.00	n. a.
F5	Continental	AG	13	1.96 ± 0.13	0.14 ± 0.06	0.02 ± 0.01	0.37 ± 0.05	0.95 ± 0.02
	Elbe	S (S_s, S_d)	10 (3, 7)	1.91 ± 0.12	0.08 ± 0.04	0.00 ± 0.00	0.35 ± 0.04	1.00 ± 0.00
F6	Continental	AG	23	1.00 ± 0.00	0.00 ± 0.00	0.00 ± 0.00	0.00 ± 0.00	n. a.
	Elbe	S (S_s, S_d)	12 (6, 6)	1.54 ± 0.11	0.54 ± 0.11	0.03 ± 0.01	0.18 ± 0.04	0.75 ± 0.09
F7	Continental	AG	15	1.04 ± 0.03	0.04 ± 0.03	0.00 ± 0.00	0.01 ± 0.01	0.48 ± 0.16
	Elbe	S (S_s, S_d)	6 (6, 0)	1.00 ± 0.00	0.00 ± 0.00	0.00 ± 0.00	0.00 ± 0.00	n. a.
F8	Continental	AG	16	1.04 ± 0.04	0.04 ± 0.04	0.00 ± 0.00	0.01 ± 0.01	0.64 ± 0.01
	Elbe	S (S_s, S_d)	25 (1, 24)	1.17 ± 0.04	0.17 ± 0.04	0.00 ± 0.00	0.04 ± 0.01	0.86 ± 0.07
F9	Continental	AG	17	1.00 ± 0.00	n. a.	0.00 ± 0.00	0.00 ± 0.00	n. a.
	Elbe	S (S_s, S_d)	1 (0, 1)	n. a.	n. a.	n. a.	n. a.	n. a.
F10	Continental	AG	14	1.03 ± 0.02	0.00 ± 0.00	0.01 ± 0.00	0.01 ± 0.00	–0.04 ± 0.00
	Danube	S (S_s, S_d)	5 (1, 4)	2.62 ± 0.18	1.59 ± 0.17	0.12 ± 0.03	0.50 ± 0.04	0.77 ± 0.06
Mean ± standard deviation		AG	18	1.36 ± 0.48	0.10 ± 0.10	0.02 ± 0.04	0.13 ± 0.18	0.68 ± 0.34
		S (S_s, S_d)	15 (3, 12)	1.64 ± 0.58	0.35 ± 0.49	0.05 ± 0.06	0.22 ± 0.20	0.81 ± 0.14

Appendix S2. Continued.

Population	Biogeogr. region River system	Fraction	*N*	A_r	pA_r	H_I	H_S	F_{IS}
**Fish storage ponds**								
S1	Continental	AG	22	1.97 ± 0.18	0.28 ± 0.09	0.05 ± 0.01	0.32 ± 0.05	0.81 ± 0.06
	Elbe	S (S_s, S_d)	27 (4, 23)	2.11 ± 0.20	0.43 ± 0.12	0.04 ± 0.01	0.35 ± 0.05	0.85 ± 0.03
S2	Continental	AG	13	1.78 ± 0.14	0.32 ± 0.09	0.02 ± 0.01	0.23 ± 0.04	0.88 ± 0.06
	Elbe	S (S_s, S_d)	8 (3, 5)	1.76 ± 0.16	0.29 ± 0.09	0.02 ± 0.01	0.23 ± 0.05	0.83 ± 0.08
S3	Continental	AG	28	1.56 ± 0.13	0.06 ± 0.03	0.02 ± 0.01	0.19 ± 0.05	0.88 ± 0.03
	Elbe	S (S_s, S_d)	9 (1, 8)	1.58 ± 0.14	0.09 ± 0.03	0.03 ± 0.01	0.21 ± 0.05	0.89 ± 0.03
S4	Continental	AG	5	2.05 ± 0.08	0.43 ± 0.13	0.04 ± 0.02	0.41 ± 0.03	0.92 ± 0.04
	Elbe	S (S_s, S_d)	5 (2, 3)	1.90 ± 0.12	0.29 ± 0.10	0.10 ± 0.03	0.30 ± 0.04	0.67 ± 0.08
S5	Continental	AG	34	1.19 ± 0.08	0.10 ± 0.05	0.01 ± 0.00	0.06 ± 0.03	0.51 ± 0.11
	Elbe	S (S_s, S_d)	16 (5, 11)	1.11 ± 0.07	0.02 ± 0.01	0.00 ± 0.00	0.05 ± 0.03	0.66 ± 0.13
S6	Continental	AG	15	1.00 ± 0.00	0.00 ± 0.00	0.00 ± 0.00	0.00 ± 0.00	n. a.
	Elbe	S (S_s, S_d)	16 (2, 14)	1.05 ± 0.04	0.05 ± 0.04	0.01 ± 0.00	0.01 ± 0.01	0.30 ± 0.10
S7	Continental	AG	30	1.16 ± 0.05	0.13 ± 0.04	0.00 ± 0.00	0.03 ± 0.01	0.86 ± 0.07
	Elbe	S (S_s, S_d)	13 (8, 5)	1.29 ± 0.09	0.26 ± 0.08	0.00 ± 0.00	0.06 ± 0.02	0.95 ± 0.03
S8	Continental	AG	27	1.38 ± 0.07	0.37 ± 0.06	0.00 ± 0.00	0.08 ± 0.01	0.98 ± 0.01
	Elbe	S (S_s, S_d)	20 (5, 15)	1.02 ± 0.02	0.01 ± 0.01	0.00 ± 0.00	0.00 ± 0.00	–0.05 ± 0.00
S9	Continental	AG	14	1.20 ± 0.07	0.05 ± 0.03	0.00 ± 0.00	0.05 ± 0.02	1.00 ± 0.00
	Elbe	S (S_s, S_d)	14 (2, 12)	1.25 ± 0.09	0.10 ± 0.05	0.01 ± 0.01	0.08 ± 0.03	0.87 ± 0.05
S10	Continental	AG	20	1.95 ± 0.11	0.69 ± 0.09	0.03 ± 0.01	0.30 ± 0.03	0.90 ± 0.03
	Elbe	S (S_s, S_d)	27 (4, 23)	1.33 ± 0.08	0.07 ± 0.03	0.02 ± 0.01	0.09 ± 0.02	0.76 ± 0.03
Mean ± standard deviation		AG	21	1.52 ± 0.39	0.24 ± 0.22	0.02 ± 0.02	0.17 ± 0.14	0.86 ± 0.14
		S (S_s, S_d)	16 (4, 12)	1.44 ± 0.38	0.16 ± 0.14	0.02 ± 0.03	0.14 ± 0.12	0.67 ± 0.32

Appendix S3. Measures of genetic diversity (mean ± standard error over loci) based on 21 microsatellite markers of populations across Europe. The values of the above-ground populations of the three habitat types (Appendix S2) were also considered for the calculation of regional means. To this aim, pA_r was calculated in relation to the complete above-ground sample across Europe (different to Appendix S2). A_r, allelic richness; pA_r, private allelic richness; H_I, observed heterozygosity; H_S, expected heterozygosity; F_{IS}, inbreeding coefficient. A_r and pA_r were determined based on 10 randomly sampled alleles per population. See Appendix S1 for a list of population codes with collection information.

Population	River system	N	A_r	pA_r	H_I	H_S	F_{IS}
**Mediterranean region**							
ES1	n. a.	12	1.09 ± 0.06	0.00 ± 0.00	0.00 ± 0.00	0.04 ± 0.02	1.00 ± 0.00
ES2	n. a.	11	2.74 ± 0.15	0.58 ± 0.13	0.03 ± 0.02	0.51 ± 0.03	0.94 ± 0.03
FR1	n. a.	11	1.47 ± 0.14	0.10 ± 0.06	0.01 ± 0.01	0.14 ± 0.04	0.92 ± 0.03
HR1	n. a.	12	1.88 ± 0.17	0.09 ± 0.06	0.00 ± 0.00	0.28 ± 0.05	1.00 ± 0.00
HR2	n. a.	18	1.14 ± 0.08	0.15 ± 0.08	0.04 ± 0.03	0.07 ± 0.04	0.35 ± 0.13
IT1	Po	9	2.91 ± 0.21	0.22 ± 0.12	0.08 ± 0.02	0.50 ± 0.04	0.81 ± 0.05
IT3	n. a.	12	2.42 ± 0.18	0.18 ± 0.08	0.01 ± 0.01	0.38 ± 0.05	0.97 ± 0.02
IT4	n. a.	11	1.66 ± 0.15	0.27 ± 0.10	0.10 ± 0.03	0.22 ± 0.05	0.45 ± 0.10
IT5	n. a.	10	1.00 ± 0.00	0.01 ± 0.01	0.00 ± 0.00	0.00 ± 0.00	n. a.
Mean ± standard deviation		12 ± 3	1.72 ± 0.68	0.18 ± 0.18	0.03 ± 0.04	0.24 ± 0.19	0.80 ± 0.26
**Pannonian region**							
AT1	Danube	12	2.74 ± 0.22	0.09 ± 0.06	0.00 ± 0.00	0.46 ± 0.05	1.00 ± 0.00
AT2	Danube	17	2.74 ± 0.18	0.04 ± 0.03	0.09 ± 0.01	0.51 ± 0.04	0.79 ± 0.05
AT3	Danube	20	2.69 ± 0.23	0.01 ± 0.01	0.05 ± 0.01	0.44 ± 0.05	0.89 ± 0.02
AT4	Danube	10	2.29 ± 0.18	0.05 ± 0.04	0.00 ± 0.00	0.40 ± 0.04	1.00 ± 0.00
HR3	Danube	9	2.78 ± 0.16	0.12 ± 0.06	0.14 ± 0.02	0.50 ± 0.04	0.69 ± 0.06
HU1	Danube	6	1.90 ± 0.14	0.05 ± 0.05	0.00 ± 0.00	0.33 ± 0.05	1.00 ± 0.00
HU2	Danube	7	2.49 ± 0.14	0.00 ± 0.00	0.02 ± 0.01	0.49 ± 0.03	0.96 ± 0.03
HU3	Danube	12	2.19 ± 0.20	0.05 ± 0.04	0.00 ± 0.00	0.33 ± 0.06	1.00 ± 0.00
HU4	Danube	8	1.99 ± 0.20	0.09 ± 0.06	0.02 ± 0.01	0.28 ± 0.05	0.87 ± 0.07
HU5	Danube	9	2.01 ± 0.19	0.00 ± 0.00	0.01 ± 0.01	0.32 ± 0.06	0.99 ± 0.01
HU7	Danube	7	2.47 ± 0.23	0.04 ± 0.04	0.05 ± 0.02	0.42 ± 0.06	0.84 ± 0.06
HU8	Danube	11	2.54 ± 0.14	0.00 ± 0.00	0.10 ± 0.02	0.48 ± 0.04	0.81 ± 0.03
HU10	Danube	8	1.93 ± 0.15	0.05 ± 0.05	0.01 ± 0.01	0.30 ± 0.05	0.99 ± 0.01

Appendix S3. Continued.

Population	River system	*N*	A_r	pA_r	H_I	H_S	F_{IS}
HU11	Danube	11	2.37 ± 0.15	0.08 ± 0.05	0.00 ± 0.00	0.40 ± 0.03	1.00 ± 0.00
HU12	Danube	7	1.73 ± 0.15	0.00 ± 0.00	0.02 ± 0.01	0.23 ± 0.05	0.88 ± 0.07
SK1	Danube	10	2.50 ± 0.17	0.00 ± 0.00	0.02 ± 0.01	0.44 ± 0.05	0.97 ± 0.02
SK2	Danube	8	1.46 ± 0.14	0.00 ± 0.00	0.00 ± 0.00	0.19 ± 0.06	1.00 ± 0.00
SK3	Danube	10	2.31 ± 0.18	0.00 ± 0.00	0.05 ± 0.01	0.43 ± 0.05	0.85 ± 0.05
SK4	Danube	9	1.87 ± 0.09	0.00 ± 0.00	0.02 ± 0.01	0.25 ± 0.03	0.92 ± 0.03
R5 (AG)	Danube	15	2.79 ± 0.19	0.08 ± 0.05	0.05 ± 0.01	0.47 ± 0.04	0.89 ± 0.03
R6 (AG)	Danube	24	3.10 ± 0.18	0.01 ± 0.01	0.29 ± 0.02	0.58 ± 0.03	0.49 ± 0.03
R7 (AG)	Danube	16	2.93 ± 0.16	0.01 ± 0.01	0.08 ± 0.01	0.54 ± 0.03	0.86 ± 0.02
R8 (AG)	Danube	15	2.99 ± 0.21	0.01 ± 0.01	0.13 ± 0.02	0.58 ± 0.03	0.77 ± 0.03
R9 (AG)	Danube	13	2.58 ± 0.14	0.05 ± 0.04	0.14 ± 0.02	0.44 ± 0.03	0.65 ± 0.06
R10 (AG)	Danube	14	3.16 ± 0.17	0.04 ± 0.03	0.06 ± 0.01	0.57 ± 0.03	0.89 ± 0.03
R11 (AG)	Danube	15	1.83 ± 0.11	0.02 ± 0.02	0.07 ± 0.02	0.26 ± 0.03	0.74 ± 0.05
Mean ± standard deviation		12 ± 4	2.30 ± 0.57	0.03 ± 0.04	0.05 ± 0.07	0.41 ± 0.11	0.88 ± 0.13
**Continental region**							
AT5	Danube	9	1.14 ± 0.07	0.04 ± 0.03	0.04 ± 0.02	0.03 ± 0.02	–0.11 ± 0.01
CH	Rhine	12	1.00 ± 0.00	0.00 ± 0.00	0.00 ± 0.00	0.00 ± 0.00	n. a.
DE1	n. a.	10	1.68 ± 0.10	0.00 ± 0.00	0.03 ± 0.01	0.25 ± 0.04	0.90 ± 0.04
DE2	Elbe	11	2.43 ± 0.15	0.00 ± 0.00	0.00 ± 0.00	0.43 ± 0.04	0.99 ± 0.01
DE3	Elbe	7	1.65 ± 0.18	0.07 ± 0.05	0.01 ± 0.01	0.22 ± 0.06	0.97 ± 0.02
DE4	Rhine	9	2.02 ± 0.20	0.02 ± 0.02	0.01 ± 0.01	0.31 ± 0.06	0.98 ± 0.01
LI1	Rhine	12	1.94 ± 0.11	0.01 ± 0.01	0.00 ± 0.00	0.25 ± 0.03	1.00 ± 0.00
LI2	Rhine	12	1.55 ± 0.12	0.00 ± 0.00	0.00 ± 0.00	0.21 ± 0.05	1.00 ± 0.00
LI3	Rhine	12	1.00 ± 0.00	0.01 ± 0.01	0.00 ± 0.00	0.00 ± 0.00	n. a.
LT	n. a.	15	1.02 ± 0.02	0.00 ± 0.00	0.00 ± 0.00	0.00 ± 0.00	–0.03 ± 0.00
R1 (AG)	Oder	15	2.46 ± 0.18	0.05 ± 0.04	0.20 ± 0.02	0.42 ± 0.05	0.52 ± 0.04
R2 (AG)	Oder	17	2.54 ± 0.15	0.00 ± 0.00	0.35 ± 0.03	0.47 ± 0.04	0.25 ± 0.05
R3 (AG)	Elbe	15	2.69 ± 0.16	0.00 ± 0.00	0.18 ± 0.02	0.48 ± 0.04	0.62 ± 0.05
R4 (AG)	Elbe	15	2.20 ± 0.14	0.06 ± 0.04	0.04 ± 0.01	0.35 ± 0.04	0.88 ± 0.03

Appendix S3. Continued.

Population	River system	N	A_r	pA_r	H_I	H_S	F_{IS}
F1 (AG)	Elbe	22	2.07 ± 0.13	0.00 ± 0.00	0.14 ± 0.02	0.38 ± 0.04	0.63 ± 0.04
F2 (AG)	Elbe	22	1.22 ± 0.07	0.00 ± 0.00	0.01 ± 0.01	0.06 ± 0.03	0.91 ± 0.03
F3 (AG)	Elbe	20	2.08 ± 0.11	0.00 ± 0.00	0.05 ± 0.01	0.41 ± 0.04	0.86 ± 0.03
F4 (AG)	Elbe	16	1.13 ± 0.05	0.03 ± 0.03	0.00 ± 0.00	0.03 ± 0.01	1.00 ± 0.00
F5 (AG)	Elbe	13	1.96 ± 0.13	0.00 ± 0.00	0.02 ± 0.01	0.37 ± 0.05	0.95 ± 0.02
F6 (AG)	Elbe	23	1.00 ± 0.00	0.00 ± 0.00	0.00 ± 0.00	0.00 ± 0.00	n. a.
F7 (AG)	Elbe	15	1.04 ± 0.03	0.00 ± 0.00	0.00 ± 0.00	0.01 ± 0.01	0.48 ± 0.16
F8 (AG)	Elbe	16	1.04 ± 0.04	0.01 ± 0.01	0.00 ± 0.00	0.01 ± 0.01	0.64 ± 0.01
F9 (AG)	Elbe	17	1.00 ± 0.00	0.00 ± 0.00	0.00 ± 0.00	0.00 ± 0.00	n. a.
F10 (AG)	Danube	14	1.03 ± 0.02	0.00 ± 0.00	0.01 ± 0.00	0.01 ± 0.00	–0.04 ± 0.00
S1 (AG)	Elbe	22	1.97 ± 0.18	0.02 ± 0.02	0.05 ± 0.01	0.32 ± 0.05	0.81 ± 0.06
S2 (AG)	Elbe	13	1.78 ± 0.14	0.00 ± 0.00	0.02 ± 0.01	0.23 ± 0.04	0.88 ± 0.06
S3 (AG)	Elbe	28	1.56 ± 0.13	0.05 ± 0.05	0.02 ± 0.01	0.19 ± 0.05	0.88 ± 0.03
S4 (AG)	Elbe	5	2.05 ± 0.08	0.00 ± 0.00	0.04 ± 0.02	0.41 ± 0.03	0.92 ± 0.04
S5 (AG)	Elbe	34	1.19 ± 0.08	0.00 ± 0.00	0.01 ± 0.00	0.06 ± 0.03	0.51 ± 0.11
S6 (AG)	Elbe	15	1.00 ± 0.00	0.00 ± 0.00	0.00 ± 0.00	0.00 ± 0.00	n. a.
S7 (AG)	Elbe	30	1.16 ± 0.05	0.00 ± 0.00	0.00 ± 0.00	0.03 ± 0.01	0.86 ± 0.07
S8 (AG)	Elbe	27	1.38 ± 0.07	0.00 ± 0.00	0.00 ± 0.00	0.08 ± 0.01	0.98 ± 0.01
S9 (AG)	Elbe	14	1.20 ± 0.07	0.00 ± 0.00	0.00 ± 0.00	0.05 ± 0.02	1.00 ± 0.00
S10 (AG)	Elbe	20	1.95 ± 0.11	0.02 ± 0.02	0.03 ± 0.01	0.30 ± 0.03	0.90 ± 0.03
Mean ± standard deviation		16 ± 7	1.59 ± 0.54	0.01 ± 0.02	0.04 ± 0.07	0.19 ± 0.17	0.73 ± 0.33
**Atlantic region**							
FR2	Loire	10	1.56 ± 0.12	0.00 ± 0.00	0.04 ± 0.01	0.18 ± 0.04	0.67 ± 0.08
FR3	Loire	9	1.20 ± 0.08	0.00 ± 0.00	0.01 ± 0.01	0.05 ± 0.02	0.92 ± 0.04
GB2	n. a.	10	1.00 ± 0.00	0.00 ± 0.00	0.00 ± 0.00	0.00 ± 0.00	n. a.
NL	Rhine	10	1.00 ± 0.00	0.00 ± 0.00	0.00 ± 0.00	0.00 ± 0.00	n. a.
Mean ± standard deviation		10 ± 1	1.15 ± 0.24	0.00 ± 0.00	0.01 ± 0.02	0.06 ± 0.09	0.80 ± 0.18

Appendix S4. Results of the linear mixed models of genetic diversity estimates (Appendices S2, S3). A_r, allelic richness; pA_r, private allelic richness; H_I, average observed heterozygosity; H_S, average expected heterozygosity under Hardy-Weinberg equilibrium; and F_{IS}, inbreeding coefficient. A_r and pA_r were determined based on 10 randomly sampled alleles per fraction and population. For populations of the three habitat types in Central Europe, pA_r was determined by pairwise comparison of the above-ground and soil fractions in each population separately. The significance of differences in least square means between categories of habitat (river, fishpond, fish storage pond) and biogeographical region (Mediterranean, Pannonian, Continental, Atlantic) is also shown (Tukey-Kramer adjustment for multiple comparisons).

**Effect**	A_r		pA_r		H_I		H_S		F_{IS}	
	X^2	P	X^2	P	X^2	P	X^2	P	X^2	P
**Populations of the three habitat types in Central Europe**										
Habitat	79.87	<0.0001	19.90	<0.0001	40.75	<0.0001	58.87	<0.0001	1.06	0.5883
Fraction	0.01	0.9246	0.13	0.7136	0.07	0.7975	0.08	0.7819	0.05	0.8277
Habitat × fraction	3.67	0.1598	9.18	0.0101	1.11	0.5745	2.11	0.3487	4.05	0.1322
**Populations across biogeographical regions in Europe**										
Region	42.63	<0.0001	49.23	<0.0001	2.26	0.5196	39.48	<0.0001	4.34	0.2273

**Comparison**	A_r		pA_r		H_I		H_S		F_{IS}	
	t	P	t	P	t	P	t	P	t	P
**Populations of the three habitat types in Central Europe**										
River vs. fishpond	7.53	<0.0001	3.61	0.0019	5.04	<0.0001	6.31	<0.0001	–0.69	0.7730
River vs. stor. pond	7.78	<0.0001	3.99	0.0006	5.82	<0.0001	6.82	<0.0001	–0.99	0.5868
Fishp. vs. stor. pond	0.14	0.9891	0.27	0.9618	0.69	0.7728	0.40	0.9155	–0.23	0.9708
**Populations across biogeographical regions in Europe**										
Med. vs. Pann.	–2.88	0.0263	5.74	<0.0001	–0.93	0.7912	–2.91	0.0243	–0.66	0.9101
Med. vs. Cont.	1.12	0.6772	6.87	<0.0001	–0.22	0.9963	0.86	0.8256	0.74	0.8826
Med. vs. Atl.	1.97	0.2077	4.55	0.0001	0.49	0.9622	1.95	0.2168	0.04	1.0000
Pann. vs. Cont.	5.90	<0.0001	1.37	0.5234	1.06	0.7155	5.56	<0.0001	2.08	0.1708
Pann. vs. Atl.	4.29	0.0003	0.97	0.7694	1.21	0.6232	4.28	0.0003	0.41	0.9759
Cont. vs. Atl.	1.45	0.4740	0.31	0.9899	0.71	0.8941	1.61	0.3808	–0.35	0.9847

Appendix S5. Private allelic richness (pA_r; mean ± standard error over loci) based on 21 microsatellite markers in two alternative selections of four arbitrarily chosen populations in each of the investigated biogeographical regions in Europe. Only the above-ground fractions were used in the populations, for which the soil seed bank has also been investigated (R2, R3, R4). pA_r was determined based on 10 randomly sampled alleles per population.

Population	pA_r
**Mediterranean region**	
ES1	0.10 ± 0.07
FR1	0.18 ± 0.08
IT3	0.34 ± 0.12
IT4	0.73 ± 0.15
Mean ± standard deviation	0.34 ± 0.28
**Pannonian region**	
HU1	0.09 ± 0.06
HU8	0.10 ± 0.04
HU12	0.05 ± 0.05
SK1	0.11 ± 0.07
Mean ± standard deviation	0.09 ± 0.03
**Continental region**	
R2 (AG)	0.00 ± 0.00
R4 (AG)	0.03 ± 0.03
CH	0.09 ± 0.06
DE1	0.17 ± 0.07
Mean ± standard deviation	0.07 ± 0.07
**Atlantic region**	
FR2	0.07 ± 0.05
FR3	0.04 ± 0.04
GB2	0.00 ± 0.00
NL	0.00 ± 0.00
Mean ± standard deviation	0.03 ± 0.03

Population	pA_r
**Mediterranean region**	
ES2	0.85 ± 0.14
HR2	0.18 ± 0.09
IT1	0.45 ± 0.16
IT5	0.31 ± 0.10
Mean ± standard deviation	0.45 ± 0.29
**Pannonian region**	
HU2	0.08 ± 0.05
HU5	0.20 ± 0.09
HU10	0.20 ± 0.09
SK2	0.05 ± 0.05
Mean ± standard deviation	0.13 ± 0.08
**Continental region**	
R3 (AG)	0.03 ± 0.03
DE2	0.04 ± 0.04
LI3	0.06 ± 0.05
LT	0.00 ± 0.00
Mean ± standard deviation	0.03 ± 0.02
**Atlantic region**	
FR2	0.01 ± 0.01
FR3	0.00 ± 0.00
GB2	0.06 ± 0.05
NL	0.00 ± 0.00
Mean ± standard deviation	0.02 ± 0.03
